# NKD1 promotes the progression and 5-FU resistance of colorectal cancer via the Wnt/β-catenin signaling

**DOI:** 10.1038/s41419-026-09077-2

**Published:** 2026-07-10

**Authors:** Lingyi Dong, Zhangquan Yang, Zelin Xu, Guotao Huang, Xuesi Yang, You Lv, Kunzhai Huang, Zhaohui Liu, Fuxing Zhang, Kewei Jiang, Zhilong Yu

**Affiliations:** 1 Department of General Surgery, The First Affiliated Hospital of Xiamen University, School of Medicine, Xiamen University, Xiamen, PR China; 2 Department of Colorectal Tumor Surgery, The First Affiliated Hospital of Xiamen University, School of Medicine, Xiamen University, Xiamen, Fujian Province PR China; 3 Department of Gastroenterological Surgery, Peking University People’s Hospital, Beijing, PR China

**Keywords:** Colon cancer, Rectal cancer

## Abstract

5-Fluorouracil (5-FU) resistance continues to pose a considerable barrier in the postoperative management of advanced colorectal cancer (CRC). This study identified Naked cuticle homolog 1 (NKD1) as a key factor associated with 5-FU resistance in CRC based on sequencing data from the GEO and TCGA databases. Elevated NKD1 expression correlated with adverse clinicopathological features and poor patient survival. Functional assays revealed that NKD1 overexpression elevated the IC50 of 5-FU, promoted tumor cell proliferation, and attenuated apoptosis in both drug-treated and untreated settings. Additionally, NKD1 promoted CRC cell migration and invasion, regulated tumor stem cell markers. Mechanistically, NKD1 stabilizes DVL protein, regulates APC and FZD7, and fosters nuclear accumulation of β-catenin, initiating transcriptional programs downstream of Wnt signaling. Silencing NKD1 synergized with 5-FU to improve therapeutic efficacy in patient-derived organoid and xenograft models. These results establish NKD1 as a key regulator of CRC malignancy and chemoresistance via Wnt/β-catenin pathway activation, supporting its potential as a dual biomarker and therapeutic target in combination regimens.

## Introduction

Colorectal cancer (CRC) represents the third most commonly diagnosed malignancy worldwide and remains a major contributor to cancer-related deaths [1]. Surgical resection with curative intent remains the cornerstone of CRC management [2]. However, many patients are diagnosed at advanced stages, which limits the potential benefits of radical surgical intervention [3, 4]. For these individuals, chemotherapy or alternative treatments are often employed to enhance clinical outcomes [5, 6]. Based on staging criteria established by the American Joint Committee on Cancer (AJCC), the risk of disease recurrence within five years postoperatively is ~30% for stage I–II patients, whereas individuals diagnosed at stage III exhibit substantially elevated recurrence rates, reaching 50–60% [7].

Consequently, adjuvant 5-fluorouracil (5-FU)-based chemotherapy is standard for reducing recurrence risk, particularly in stage III and high-risk stage II patients [8]. While most patients benefit from chemotherapy, a subset fails to respond, experiencing chemotherapy resistance [9]. These non-responders often undergo several ineffective treatment cycles and may suffer more from side effects than disease progression [10]. Over the years, various strategies have been incorporated into 5-FU-based therapies [11, 12], but resistance remains a significant challenge, reducing treatment efficacy in patients with refractory CRC.

Naked cuticle homolog 1 (NKD1), initially identified in Drosophila melanogaster, is recognized as an inhibitor of the Wnt signaling pathway. Typically, its expression is reduced in a range of tumors, where it functions by binding to DVL1 to suppress the Wnt/β-catenin signaling cascade. Paradoxically, NKD1 exhibits elevated expression levels in colorectal cancer. The specific role and underlying mechanisms of NKD1 in conferring resistance to 5-FU treatment in colorectal cancer remain unexplored, warranting further investigation.

This study employed integrated bioinformatics analysis using public databases such as TCGA and GDSC to identify genes critical for CRC progression and 5-FU resistance. Experimental validation confirmed that NKD1 is a potential predictor of chemotherapy resistance and poor prognosis in CRC. Furthermore, clinical correlations and data from patient-derived organoids (PDOs) and patient-derived xenografts (PDXs) models support NKD1 as a candidate dual biomarker for predicting 5-FU responsiveness and disease prognosis in CRC.

## Methods

### Patients and clinical specimens

Between 2018 and 2019, 75 paired CRC and nearby normal tissue samples were gathered from The First Hospital Affiliated to Xiamen University. Following immediate fixation in formalin, samples were paraffin-embedded and utilized to construct tissue microarrays (TMA), of which 70 paired specimens were ultimately incorporated. In 2022 and 2023, an additional 60 fresh specimens were collected from patients undergoing surgical resection for primary CRC. All specimens were independently reviewed and categorized by two board-certified pathologists in accordance with the classification standards established by the Union for International Cancer Control (UICC). No patient received neoadjuvant radiotherapy or chemotherapy. All individuals involved in the study gave their informed consent, and the protocol was approved by the hospital’s ethics review board.

### Cell lines culture and plasmid construction

Human CRC cell lines including DLD1, HCT15, HCT8, HCT116, LoVo, RKO, SW480, SW620, SW1116, as well as normal colon epithelial cell lines NCM460 and FHC, and 293 T cells were obtained from the Cell Bank of the Chinese Academy of Sciences in Shanghai, China. In a humid environment with 5% CO₂ at 37 °C, cells were maintained in DMEM supplemented with 10% fetal bovine serum (FBS) and 1% penicillin-streptomycin. To develop resistance to 5-fluorouracil, HCT8 cells were chronically exposed to increasing concentrations of 5-FU (Tsingke, China) up to 5 µg/mL, and resistance was verified via IC₅₀ analysis.

To modulate NKD1 expression levels, constructs encoding shRNA and overexpression vectors were synthesized. The sequences of shRNAs and siRNAs are detailed in Supplementary Tables S2, 3. Additionally, the primers for NKD1 CDS amplification and the full-length sequence of the inserted fragment are summarized in Supplementary Table S4.

### Assessment of CCK8 assay, clonogenic potential, DNA synthesis, and apoptosis

For the CCK8 assay, 2000 cells were placed in each well of 96-well plates and cultured at 37 °C under standard incubation conditions (37 °C, 5% CO_2_, humidified atmosphere). After 24 h, 10 μL of CCK8 reagent (Yeasen, China) was introduced, and the cells were allowed to react for 4 h. The absorbance at 450 nm was used to reflect metabolic activity. CCK8 assays were conducted every 24 h for five days. Clonogenic capacity was assessed by seeding 600 cells into each well of a 6-well plate. After 14 days in culture, colonies were fixed with 4% paraformaldehyde and stained for 15 min with 0.1% crystal violet. The surplus stain was eliminated through washing, and the number of colonies was counted using a light microscope. The EdU assay was performed using the EdU Cell Proliferation Kit with Alexa Fluor 488 from Beyotime Biotechnology, China. For cell apoptosis assays, cells were harvested and placed in a binding buffer enriched containing Annexin V (5 μL) and PI (10 μL) for 15 min, avoiding light exposure. Subsequently, apoptosis analysis was conducted utilizing a FACS Celesta flow cytometer. All experiments were executed in triplicate.

### Cell wound healing and transwell migration/invasion assays

To evaluate cell movement, cells were grown in 6-well plates to form a confluent monolayer, and then a wound-healing assay was carried out. To create a straight-line scratch, a sterile 20 μL pipette tip was utilized. Loose cells were gently washed away, and the cultures were kept in a serum-free medium to reduce cell proliferation. The process of wound closure was tracked by capturing images at 0 and 24 h after scratching. The migratory capacity was assessed by measuring wound width at 24 h and calculating the percentage of closure relative to the initial wound area.

For the transwell experiments, 2 × 10⁴ cells were mixed in 200 μL of serum-free DMEM and seeded into the upper chamber of transwell inserts with an 8.0 μm pore size (Corning, USA). In invasion assays, the upper membranes were coated with Matrigel from BD Biosciences, USA, whereas uncoated chambers were utilized for migration. The lower compartments contained 600 μL of DMEM with 10% FBS, which functioned as a chemoattractant. Following a 24-hour incubation period the cells remaining on the upper surface were cautiously removed. The cells that moved through the membrane were fixed with 4% paraformaldehyde for 30 min and stained using 0.1% crystal violet. Using a microscope, stained cells on the bottom surface were viewed, and five fields per insert were randomly picked for imaging and analysis.

### Quantitative real-time PCR (qRT-PCR) and Western blotting

Total RNA was extracted from cultured cells and tissue samples using TRIzol reagent (TaKaRa, Japan) following the standard procedure. Afterward, Strand cDNA Synthesis SuperMix (Yeasen, China) was used to reverse transcribe the RNA into cDNA. RNA expression was measured using SYBR Green Master Mix from Yeasen, China, through qRT-PCR on a ViiA 7 Fast Real-Time PCR system by Applied Biosystems, USA. Using the 2^−ΔΔCt^ method, relative gene expression was assessed with GAPDH as the normalization control. Details of the primer sequences are provided in Supplementary Table S1.

Western blotting was carried out following established procedures [13]. Protein extracts were separated using SDS-PAGE, transferred onto PVDF membranes, and then incubated with primary antibodies targeting NKD1, β-catenin, CD44, SOX2, LGR5, Axin1, Dvl1, APC, and FZD7 (all at 1:1000; Affinity, Abcam, or Proteintech). β-actin (1:2000; ABclonal, China) served as the loading control. For visualization, HRP-conjugated secondary antibodies against rabbit or mouse (1:5000; ABclonal, China) were utilized.

### Coimmunoprecipitation (Co-IP)

For the Co-immunoprecipitation (CoIP) assay, HCT116 cells stably expressing empty vector (Vector group) or NKD1 overexpression construct (OE-NKD1 group) were cultured to 70~80% confluence, washed twice with pre-cooled PBS, then lysed in ice-cold RIPA buffer (supplemented with protease inhibitor cocktail and 1 mM PMSF) for 30 min on ice; the lysate was centrifuged at 12,000 × *g* for 15 min at 4 °C, and the supernatant (total protein) was collected and quantified via BCA assay to ensure equal loading. Next, 1 mg of total protein (adjusted to 1 mL with RIPA buffer) per sample was incubated with 2 μg of anti-Dvl1/anti-β-catenin antibody (or normal IgG as negative control) at 4 °C with gentle rotation overnight, followed by addition of 50 μL pre-washed Protein A/G agarose beads and further 4-h incubation at 4 °C; the bead-protein complexes were collected by centrifugation (500 × *g*, 5 min, 4 °C), washed 5 times with pre-cooled washing buffer, then mixed with 2× SDS loading buffer and boiled at 95 °C for 10 min to elute immunoprecipitated proteins (supernatant collected post-centrifugation). A 5% aliquot of the original total protein (Input) and IP samples were separated by 10% SDS-PAGE, transferred onto PVDF membranes, blocked with 5% non-fat milk in TBST for 1 h, incubated with primary antibodies (anti-Dvl1, anti-β-catenin, anti-NKD1, or anti-β-actin) at 4 °C overnight, then with HRP-conjugated secondary antibody for 1 h at room temperature; protein bands were visualized via ECL detection.

### Immunohistochemistry (IHC)

The CRC tissue microarrays were dewaxed, rehydrated, and subjected to antigen retrieval using a 10 mM sodium citrate buffer (pH 6.0) at boiling temperature for 5 min. To inactivate endogenous peroxidase, 3% hydrogen peroxide was applied. Overnight incubation of the sections at 4 °C was performed using primary antibodies for NKD1 (1:500), β-catenin (1:800), Bax (1:800), Ki-67 (1:300), Bcl-2 (1:500; all from Abcam), and FZD7 (1:200; Affinity). Following a 30-min incubation with the secondary antibody at room temperature, DAB was used for visualization, and hematoxylin was applied for counterstaining.The slides were dehydrated and sealed. The staining evaluation was conducted independently by two blinded observers, who scored each sample according to the percentage of positive cells and the intensity of the staining.

### Tissue preparation and organoid culture

Tissue samples were transferred into sterile 60 mm dishes, minced into small pieces, and rinsed thrice with ice-cold PBS. After washing with 10 mL Advanced DMEM/F12, samples were subjected to enzymatic digestion using 2 mg/mL collagenase at 37 °C for 12 h. The digested material was filtered through a 70 μm cell strainer, rinsed twice in a medium containing 2% fetal calf serum, and then centrifuged at 400 rcf for 4 min. Single-cell suspensions were subsequently pelleted at 300 × *g*, 4 °C for 3 min. A mixture containing 1.1 mL of complete colorectal organoid culture medium and 120 μL of pre-chilled Matrigel was prepared and seeded into ultra-low attachment plates (Costar). After 30 min of incubation at 37 °C to allow matrix polymerization, 300 μL of growth medium was added. Cells were embedded in reduced-growth-factor Matrigel and overlaid with 500 μL of human organoid medium (HOM). Medium changes were performed every two days or as needed.

### Multiplex fluorescent IHC staining

CRC organoid sections (5 μm, formalin-fixed, paraffin-embedded) were mounted on slides, baked at 65 °C for 2 h, dewaxed in xylene (15 min ×2), followed by gradient rehydration through 100%, 95%, and 75% ethanol. Slides were washed with water, and antigen retrieval was conducted using a sodium citrate buffer with a pH of 6.0. Multiplex immunofluorescence staining was carried out with the Opal™ Multiplex IHC kit (Akoya). Primary antibodies and fluorophores were as follows: NKD1 (1:250, Opal 520), Ki67 (1:250, Opal 570), CEA (1:400, Opal 620), and Ep-CAM (1:250, Opal 690), all from Abcam. Nuclear staining was performed using DAPI. Slides were mounted using ProLong™ Diamond Antifade (ThermoFisher) and sealed with coverslips.

### Subcutaneous xenograft and PDX models

For subcutaneous tumorigenesis, 5× 10^6^ CRC cells suspended in 150 μL PBS were administered into the right flanks of 4-week-old male BALB/c nude mice. Starting from day 10 post-implantation, 5-fluorouracil (50 mg/kg) was administered intraperitoneally twice per week. Tumor dimensions were recorded weekly. Following a 4-week treatment period, the mice were euthanized, and the tumors were removed, weighed, and prepared for histological analysis.

For PDX establishment, tumor tissues resected from CRC patients were implanted into 4-week-old male NOD/SCID (NCG) mice. Once tumor volumes reached ~150 mm³, all mice were randomized into three groups (*n* = 5 per group): sh-NC group, sh-NKD1#1 and sh-NKD1#2 group and treated with intratumor injection of appropriate lentivirus once every two weeks. Tumor progression was tracked weekly. After 5 weeks, animals were sacrificed, and tumors were harvested for volume, protein extraction and histopathological assessment.

All experimental procedures were conducted in accordance with the approved animal handling protocols of the Laboratory Animal Center of Xiamen University and received approval from the Animal Use and Management Committee of Youshu Life Technology (Shanghai) Co., Ltd. [YS-m202408003].

### Statistical analysis

For all quantitative data, the χ2 or Fisher’s exact test was employed. Correlation coefficients were determined by Pearson’s test (r, P). The analysis of continuous data was performed with the Student’s *t* test or the Mann–Whitney U test, depending on distribution. The Kaplan–Meier method was used to assess survival outcomes, and comparisons were made using log-rank tests. A two-tailed *P* value < 0.05 was deemed statistically significant. The GraphPad Prism 9.0 and SPSS 28.0 software were utilized for conducting statistical analyses.

## Results

### Expression of NKD1 and its clinical significance

**Fig. 1:**
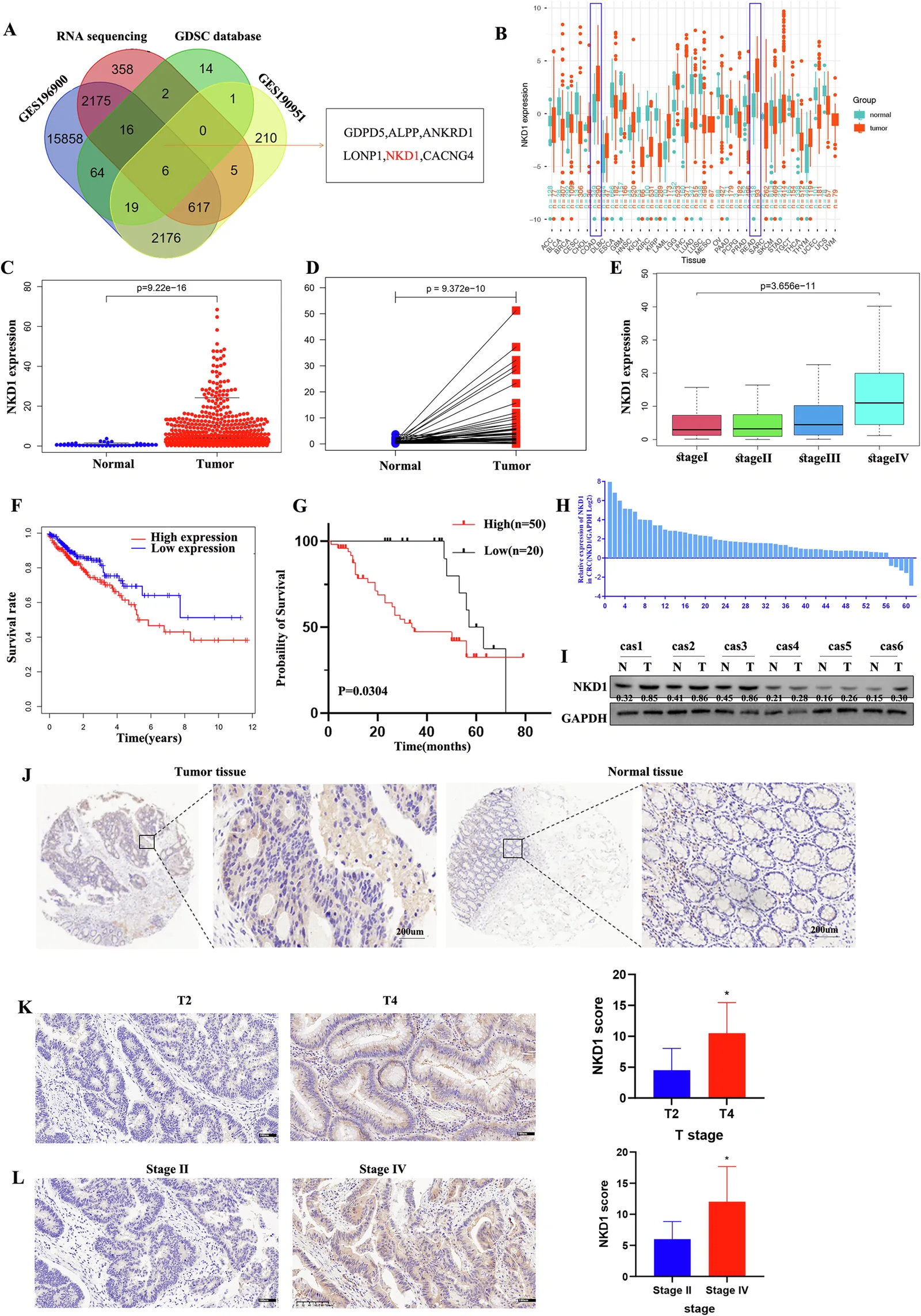
Expression of NKD1 and its clinical significance. **A** Cross-database identification of 5-FU resistance biomarkers in colorectal cancer. **B** Pan-cancer expression pattern of NKD1 from the TCGA and Gtex database. **C**, **D** Overall and paired expression of NKD1 in colorectal cancer tissues. **E** Expression of NKD1 in different stages of colorectal cancer. **F** Comparison of overall survival (OS) between patients with high and low NKD1 expression in the TCGA database. **G** Survival analysis of colorectal cancer patients stratified by NKD1 expression levels. **H** NKD1 mRNA expression in 60 colorectal cancer tissues. **I** NKD1 protein expression in six pairs of colorectal cancer and corresponding normal tissues. **J** Representative images of NKD1 expression in colorectal cancer and corresponding adjacent normal tissues (bar = 200 μm). **K** Comparison of NKD1 expression in T2 and T4 colorectal cancer tissues (bar = 100 μm). **L** Comparison of NKD1 expression levels in stage II and stage IV colorectal cancer tissues (bar = 100 μm). Results are expressed as mean ± standard deviation (SD). * indicates *P* < 0.05; ** indicates *P* < 0.01.

Integrated analysis of GEO (comparing 5-FU-resistant and parental cell lines) and GDSC databases identified six candidate genes associated with 5-FU resistance, including NKD1 (Fig. 1A). GEPIA database analysis showed that among the six DEGs, only NKD1 was differentially expressed in both colon and rectal cancers (Supplementary Fig. S1A), leading to its selection for subsequent validation. Pan-cancer analysis via TCGA and Gtex revealed elevated NKD1 expression in CRC and low-grade glioma (Fig. 1B). Further analysis of TCGA data confirmed that NKD1 expression is significantly higher in CRC tissues (Fig. 1C, D). Specifically, NKD1 expression was higher in stage IV patients than in stages I and II (Fig. 1E), and the high-expression group had significantly lower overall survival (OS) rates than the low-expression group (Fig. 1F, G). To validate these findings, qRT-PCR and western blotting demonstrated increased NKD1 expression in CRC tissues and cell lines (Fig. 1H, I, Supplementary Fig. S1B, C). IHC results showed that NKD1 was mainly localized in the nucleus and cytoplasm (Fig. 1J), and the expression level of NKD1 in colorectal cancer tissues was significantly higher than that in adjacent normal tissues. Tissue microarray analysis (Table 1) revealed that NKD1 expression correlated with the T (Fig. 1K), N, and tumor differentiation and UICC stages (Fig. 1L). These data suggest that NKD1 may function as a key oncogenic factor and holds potential as a diagnostic and therapeutic target in CRC.

**Table 1:** Correlations between NKD1 expression and clinicopathological characteristics of CRC patients (*n* = 70).

**Characteristics**	***N***	**NKD1 expression**		***χ***^*2*^	***P*** **value**
		**Low**	**High**		
Age					
<65	33	7	26	8.258	**0.004**
≥65	37	13	24		
Gender					
Male	40	12	28	2.382	0.123
Female	30	8	22		
T stage					
T1 + T2 + T3	35	10	25	5.600	**0.018**
T4	35	10	25		
N stage					
N0 + N1	32	17	15	6.722	**0.009**
N2 + N3	38	3	35		
UICC stage					
I+II	47	7	40	7.418	**0.024**
III + Ⅳ	23	13	10		
Neural invasion					
+	38	12	26	3.361	0.067
-	32	8	24		
Vascular invasion					
+	39	9	30	0.250	0.617
-	31	11	20		
Histological differentiation					
Well differentiated	19	11	8	18.132	**<0.001**
Moderately/Poorly differentiated	51	9	42		
Tumor diameter					
≤3 cm	25	10	15	2.700	0.1003
>3 cm	45	10	35		
Tumor or Normal tissue					
Tumor	70	20	50	11.667	**<0.001**
Normal tissue	70	40	30		

Bold font indicates a statistically significant association.

### NKD1 enhanced CRC cell growth and metastasis

**Fig. 2:**
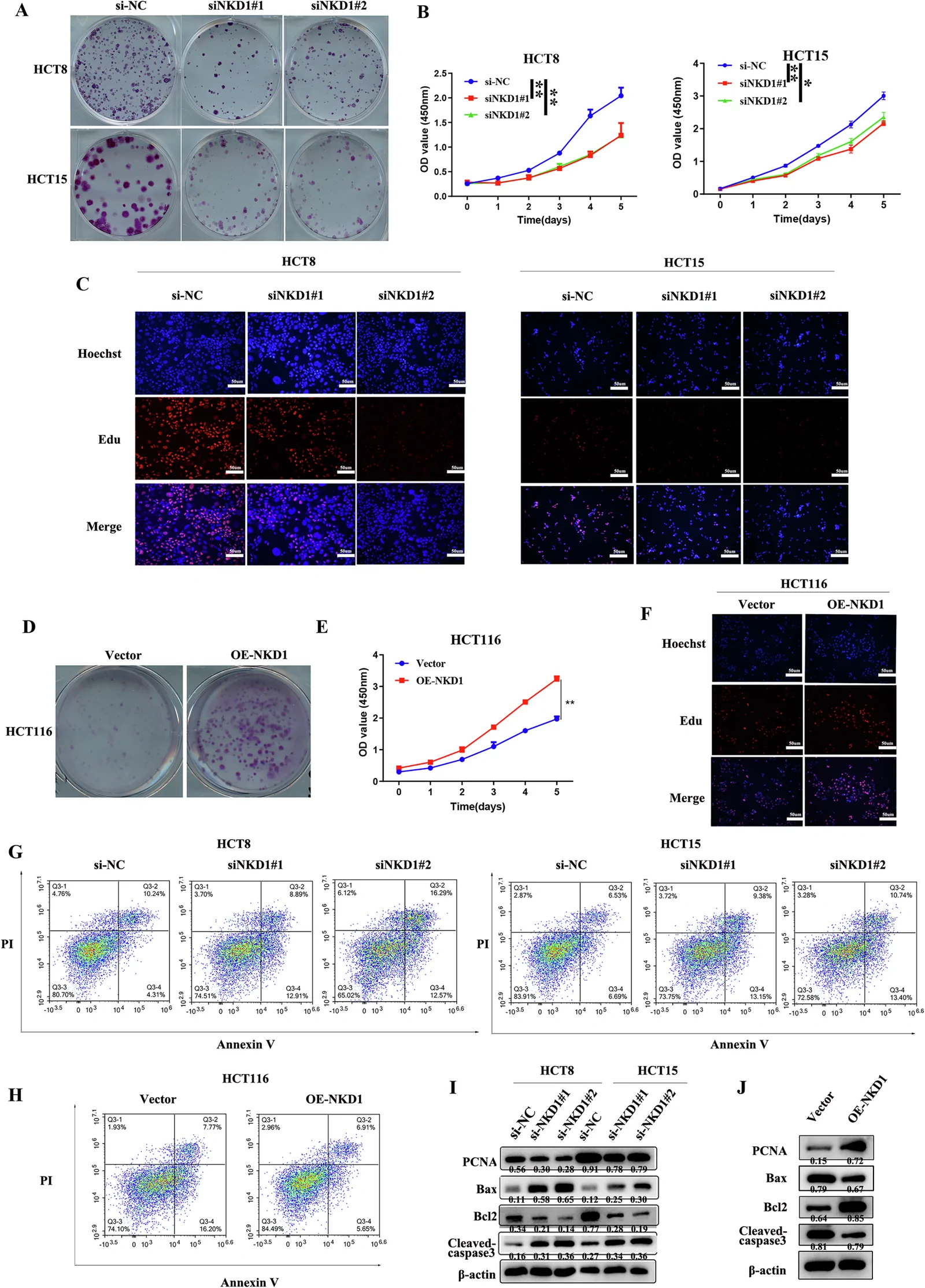
NKD1 promotes malignant proliferation and inhibits apoptosis of colorectal cancer cells in vitro. **A** Colony formation assay assessing cell proliferation after NKD1 knockdown in HCT8 and HCT15 cells. **B** CCK-8 assay assessing cell proliferation after NKD1 knockdown in HCT8 and HCT15 cells. **C** Representative images and quantitative analysis of EdU assays from HCT8 and HCT15 cells treated with NKD1 shRNA or control for 48 h. Scale bar, 50 μm. **D** Colony formation assay assessing cell proliferation after NKD1 overexpression in HCT116 cells. **E** CCK-8 assay assessing cell proliferation after NKD1 overexpression in HCT116 cells. **F** Representative images and quantitative analysis of EdU assays from HCT116 cells treated with NKD1 overexpression or control for 48 h. Scale bar, 50 μm. **G** Comparison of apoptotic cell numbers between NKD1-knockdown and control cells in HCT8 and HCT15 cells via Annexin V-PI assays. **H** Comparison of apoptotic cell numbers between NKD1-overexpression and control cells in HCT116 cells via Annexin V-PI assays. **I** Protein expression of apoptosis-related genes in HCT8 and HCT15 cells after NKD1 downregulation. **J** Protein expression of apoptosis-related genes in HCT116 cells after NKD1 upregulation. Results are expressed as mean ± standard deviation (SD). * indicates *P* < 0.05; ** indicates *P* < 0.01.

**Fig. 3:**
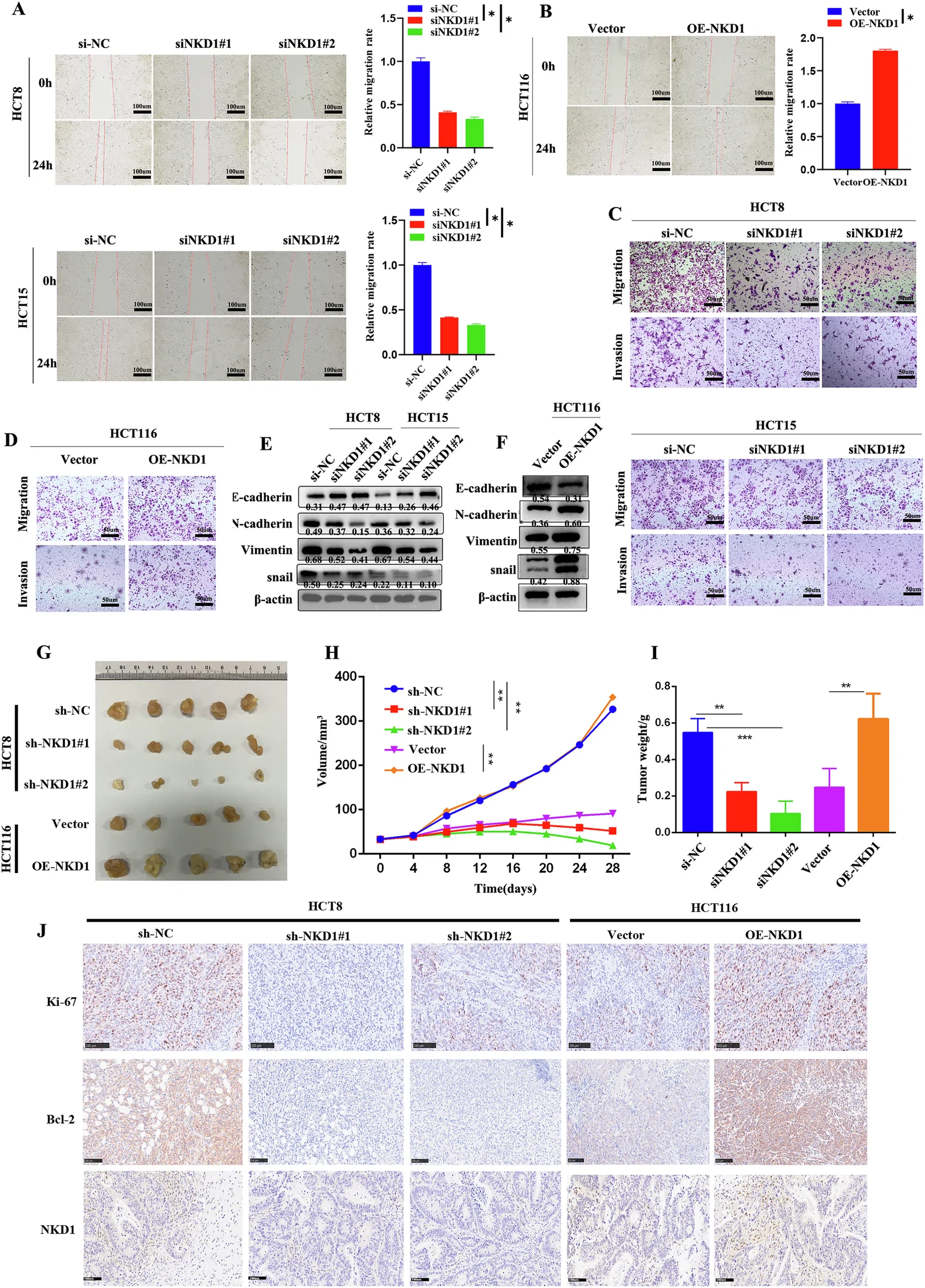
NKD1 promotes in vitro migration and invasion of colorectal cells and promotes in vivo proliferation. **A**, **B** Wound healing assays evaluating the migration abilities of CRC cells after altering NKD1 levels. Scale bar, 100 μm. **C**, **D** Transwell assays demonstrated the migration capabilities of CRC cells following changes in NKD1 levels. Scale bar, 50 μm. **E** Representative images of EMT marker expression from HCT8 and HCT15 cells treated with NKD1 shRNA or control for 48 h. **F** Representative images of EMT marker expression from HCT116 cells treated with NKD1 overexpression or control for 48 h. **G** Representative images of harvested tumors from different groups on Day 28. The sh-NC, sh-NKD1#1 and sh-NKD1#2 groups were constructed with HCT8 cell-derived xenografts, while the Vector and OE-NKD1 groups were based on HCT116 cell-derived xenografts. **H** Tumor volume measurements across different treatment groups (*n* = 5 per group). **I** Tumor weight quantification across different treatment groups (*n* = 5 per group). **J** NKD1 is associated with upregulated expression of Ki67 and Bcl-2, with representative images of IHC results. Scale bar, 100 μm. Results are expressed as mean ± standard deviation (SD). * indicates *P* < 0.05; ** indicates *P* < 0.01; *** indicates *P* < 0.001.

To elucidate the functional role of NKD1 in CRC, expression was modulated using siRNA-mediated knockdown and plasmid-driven overexpression constructs (si-NKD1, OE-NKD1), with qRT-PCR and western blotting confirming transfection efficiency (Supplementary Fig. S2A–F). Functional assays, including colony formation, CCK-8, and EdU staining, revealed that silencing NKD1 markedly suppressed the proliferative capacity of HCT8 and HCT15 cells (Fig. 2A–C, Supplementary Fig. S2G, H), while forced NKD1 expression significantly enhanced proliferation in HCT116 cells (Fig. 2D–F, Supplementary Fig. S2I, J). Apoptosis assays revealed that NKD1 downregulation promoted apoptosis in HCT8 and HCT15 cells (Fig. 2G, Supplementary Fig. S2K), while NKD1 upregulation inhibited apoptosis in HCT116 cells (Fig. 2H, Supplementary Fig. S2L). In HCT8 and HCT15 cells, NKD1 knockdown significantly increased Bax and Cleaved-caspase3 expression and decreased PCNA and Bcl2 levels compared to the si-NC group (Fig. 2I). Conversely, the opposite phenomenon was observed when NKD1 was overexpressed (Fig. 2J). NKD1 also regulated metastatic phenotypes. Its depletion impaired cell migration and invasion in wound healing and Transwell assays, while overexpression enhanced these capabilities (Fig. 3A–D, Supplementary Fig. S2M, N). Furthermore, NKD1 knockdown increased the epithelial marker E-cadherin and decreased the mesenchymal markers N-cadherin and Snail, consistent with a suppression of epithelial-mesenchymal transition (EMT); again, overexpression had the converse effect (Fig. 3E, F). Together, these findings suggest that NKD1 facilitates tumor cell proliferation, survival, and metastatic behavior in vitro.

We next assessed the role of NKD1 in tumor growth using subcutaneous xenograft models in nude mice. Consistent with the in vitro data, NKD1 knockdown (shNKD1) in HCT8 cells significantly inhibited tumor growth, resulting in smaller, lighter tumors compared to the sh-NC control. Conversely, NKD1 overexpression (OE-NKD1) in HCT116 cells accelerated tumor growth, yielding larger tumors than the vector control (Fig. 3G–I). IHC staining of tumor sections confirmed that NKD1 knockdown reduced the expression of NKD1, Ki-67, and Bcl-2, while NKD1 overexpression led to their upregulation (Fig. 3J). Thus, NKD1 is a potent driver of CRC tumor growth in vivo.

### NKD1 promoted 5-FU resistance by reducing cell apoptosis in CRC cells

**Fig. 4:**
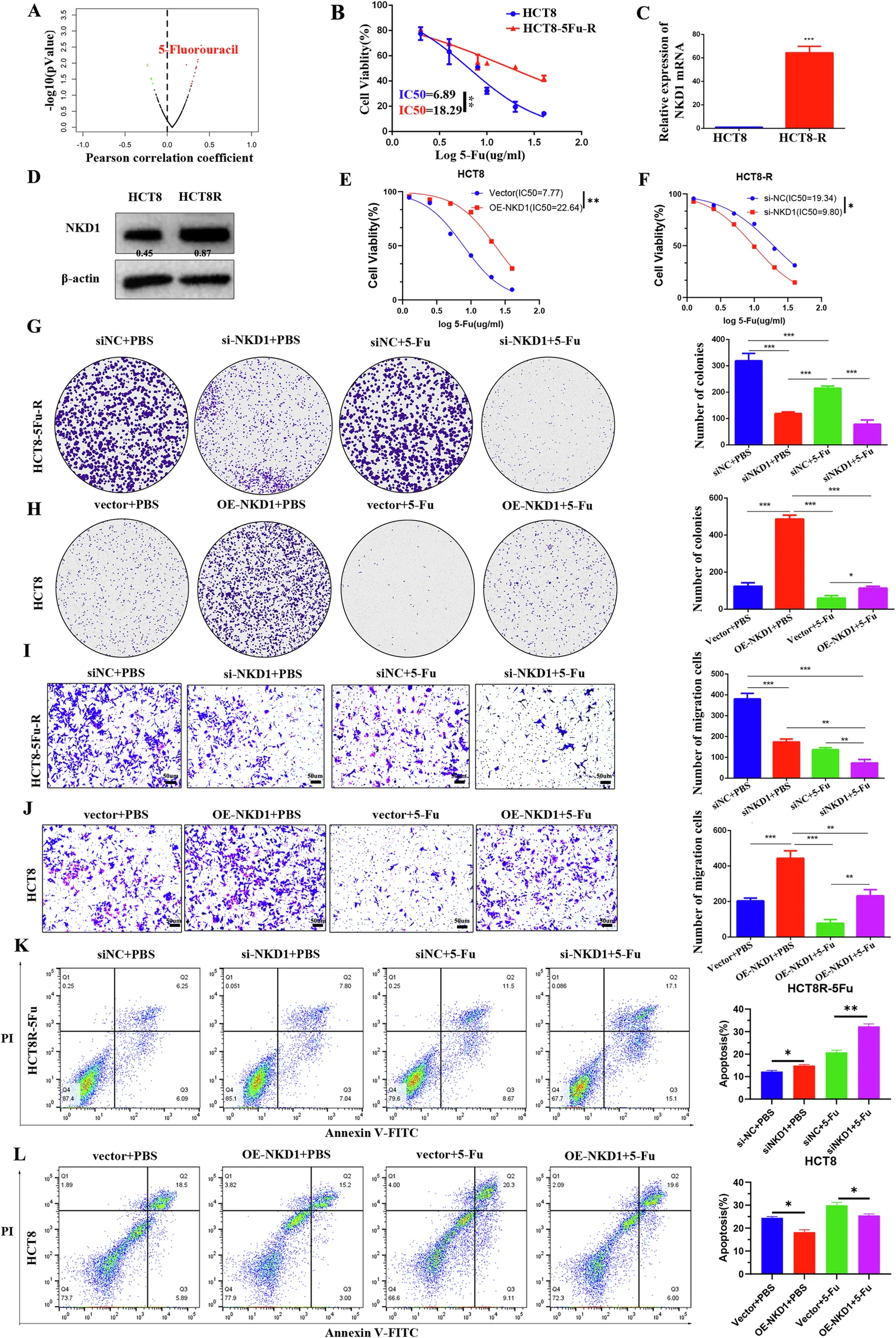
NKD1 promotes 5-FU resistance by promoting cell proliferation and migration and inhibiting apoptosis. **A** The GDSC database predicts a positive correlation between NKD1 and 5-FU drug resistance. **B** IC50 comparison between HCT8R-5FU and HCT8 cell lines, confirming successful construction of the HCT8 drug-resistant strain. **C** NKD1 mRNA expression in HCT8 and HCT8R cells. **D** NKD1 protein expression in wild-type HCT8 and drug-resistant HCT8R cells. **E** IC50 profile of NKD1 upregulation in HCT8 cells with 5-FU treatment. **F** IC50 profile of NKD1 downregulation in HCT8R cells compared with control 5-FU treatment. **G** Colony formation analysis after si-NKD1 transfection in HCT8R-5FU cells with or without 5-FU treatment. **H** Colony formation analysis in HCT8 cells transfected with or without si-NKD1 treatment with or without 5-FU. **I** Migration analysis after transfection of si-NKD1 in HCT8R-5FU cells with or without 5-FU treatment. Scale bar, 50 μm. **J** Migration analysis after transfection of si-NKD1 in HCT8 cells with or without 5-FU treatment (scale bar = 50 μm). **K** Apoptosis analysis of HCT8R-5FU cells after si-NKD1 transfection with or without 5-FU treatment. **L** Apoptosis analysis of HCT8 cells after NKD1 overexpression with or without 5-FU treatment. Results are expressed as mean ± standard deviation (SD). ns indicates *P* > 0.05; * indicates *P* < 0.05; ** indicates *P* < 0.01; *** indicates *P* < 0.001.

To assess the association between NKD1 expression and drug response, gene expression and drug sensitivity profiles from the GDSC database were analyzed. Pearson correlation revealed a positive association between NKD1 levels and resistance to 5-FU (Fig. 4A). To explore the impact of NKD1 on 5-FU treatment, we developed a 5-FU-resistant HCT8 cell line (HCT8R) by prolonged exposure to low concentrations of 5-FU. IC50 determination confirmed resistance, with HCT8R cells displaying a markedly elevated IC50 (18.29 μg/mL) compared to parental HCT8 cells (6.89 μg/mL) (Fig. 4B). These results confirm the successful establishment of 5-FU resistance in HCT8 cells. qRT-PCR and western blot analyses showed higher NKD1 expression in HCT8R cells (Fig. 4C, D). Gain- and loss-of-function experiments confirmed a direct role for NKD1 in modulating 5-FU response. Overexpressing NKD1 in parental HCT8 cells increased the IC50, whereas silencing NKD1 in HCT8R cells sensitized them, lowering the IC50 (Fig. 4E, F). Furthermore, NKD1 knockdown in resistant cells reduced colony formation and migration and increased apoptosis, even in the absence of 5-FU (Fig. 4G, I, K). In addition, NKD1 knockdown in HCT8-5FU-R cells restored their sensitivity to 5-FU. In wild-type colorectal cancer (CRC) cell lines, 5-FU exerted a stronger inhibitory effect on cell proliferation than si-NKD1-mediated NKD1 silencing (Supplementary Fig. S3). Conversely, NKD1 overexpression in sensitive cells promoted colony formation and migration and suppressed apoptosis (Fig. 4H, J, L). Collectively, these findings demonstrated that NKD1 contributes to 5-FU resistance in CRC cells.

### NKD1 maintained the stemness of CRC cells

**Fig. 5:**
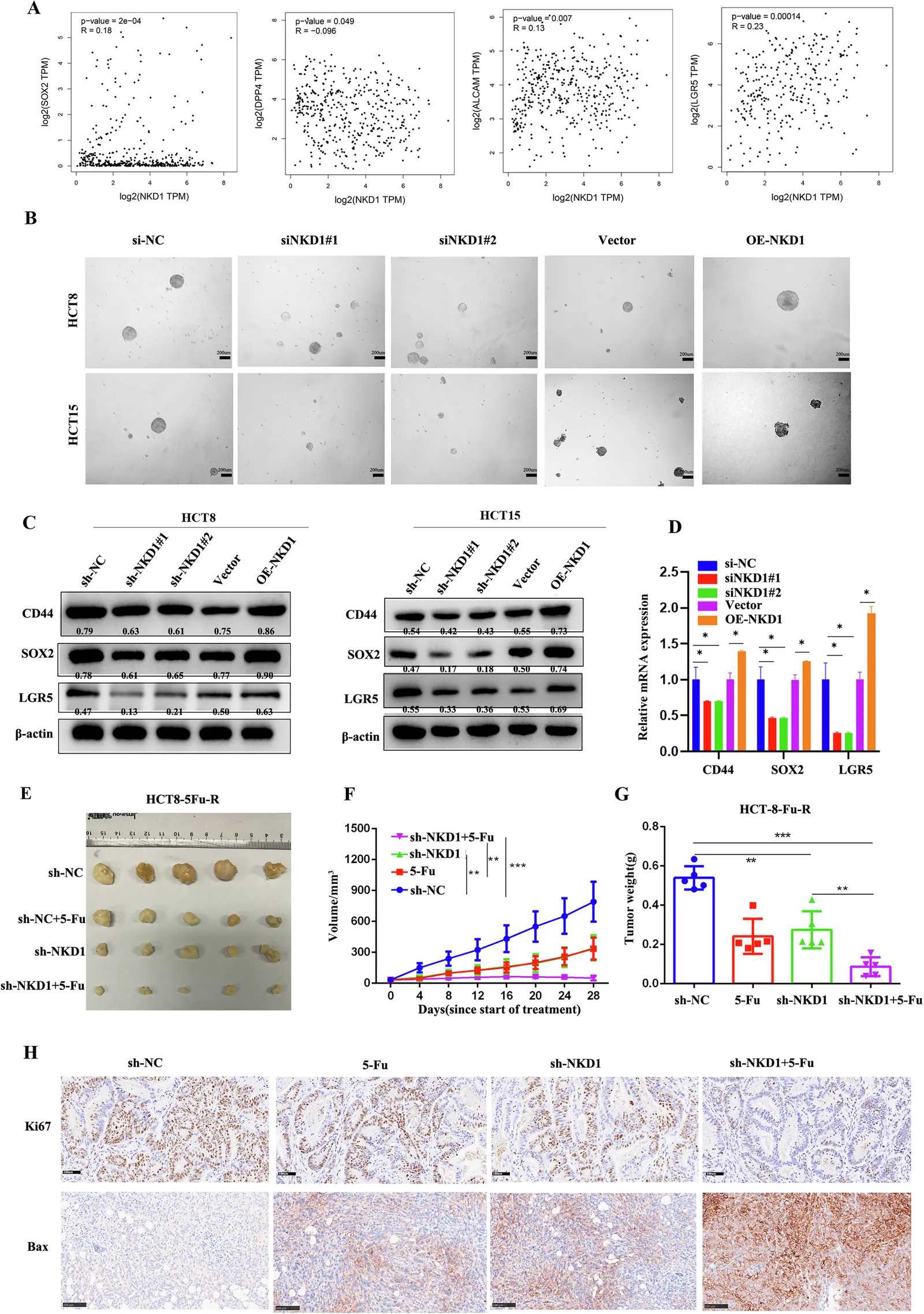
NKD1 maintained the stemness of CRC cells in vitro and in vivo. **A** Correlation between NKD1 expression and stem cell markers CD26, CD166, SOX2, and LGR5 from the GEPIA database. **B** Changes in spheroid formation after upregulation and downregulation of NKD1 in HCT8 and HCT15 cells (bar = 200 μm). **C** Changes in CD44, SOX2, and LGR5 protein expression after NKD1 upregulation and downregulation in HCT8 and HCT15 cells. **D** Changes in CD44, SOX2, and LGR5 mRNA expression after NKD1 upregulation and downregulation in HCT8 and HCT15 cells. **E** Tumor treatment using HCT8R-5FU with or without 5-FU in each group (*n* = 5). **F** Tumor volume growth trend in shNC, 5-FU, sh-NKD1, and shNKD1 + 5-FU groups. **G** Final tumor weight in different treatment groups. **H** Expression of Ki67 and Bax in different treatment groups (bar = 100 μm). Results are expressed as mean ± standard deviation (SD). * indicates *P* < 0.05; ** indicates *P* < 0.01; *** indicates *P* < 0.001.

Given that chemoresistance is a hallmark of cancer stem cells (CSCs), we examined whether NKD1 influences stemness in CRC cells. Analysis of TCGA CRC data showed a positive correlation between NKD1 transcript levels and established CSC markers, including SOX2, DPP4, CD166, and LGR5 (Fig. 5A). Functionally, NKD1 knockdown impaired spheroid-forming capacity, while its overexpression enhanced it (Fig. 5B, Supplementary Fig. S2O). At the molecular level, protein expression of the stemness markers CD44, SOX2, and LGR5 correlated positively with NKD1 levels; knockdown reduced their expression, and overexpression increased it (Fig. 5C, D). These findings suggest that NKD1 plays a critical role in maintaining the stemness of tumor cells.

The impact of NKD1 on 5-FU resistance was examined in xenograft mouse models. Knockdown of NKD1 decreased tumor volume and weight under 5-FU treatment (Fig. 5E–G). We also detected the expression of Ki67 (a biomarker of cell proliferation) and Bax (a biomarker of apoptosis) in tumor tissue slices (Fig. 5H). NKD1 knockdown reduced both markers under 5-FU treatment, indicating that NKD1 promotes 5-FU resistance in CRC cells in vivo.

### Validation of NKD1-mediated 5-FU resistance in CRC organoids

**Fig. 6:**
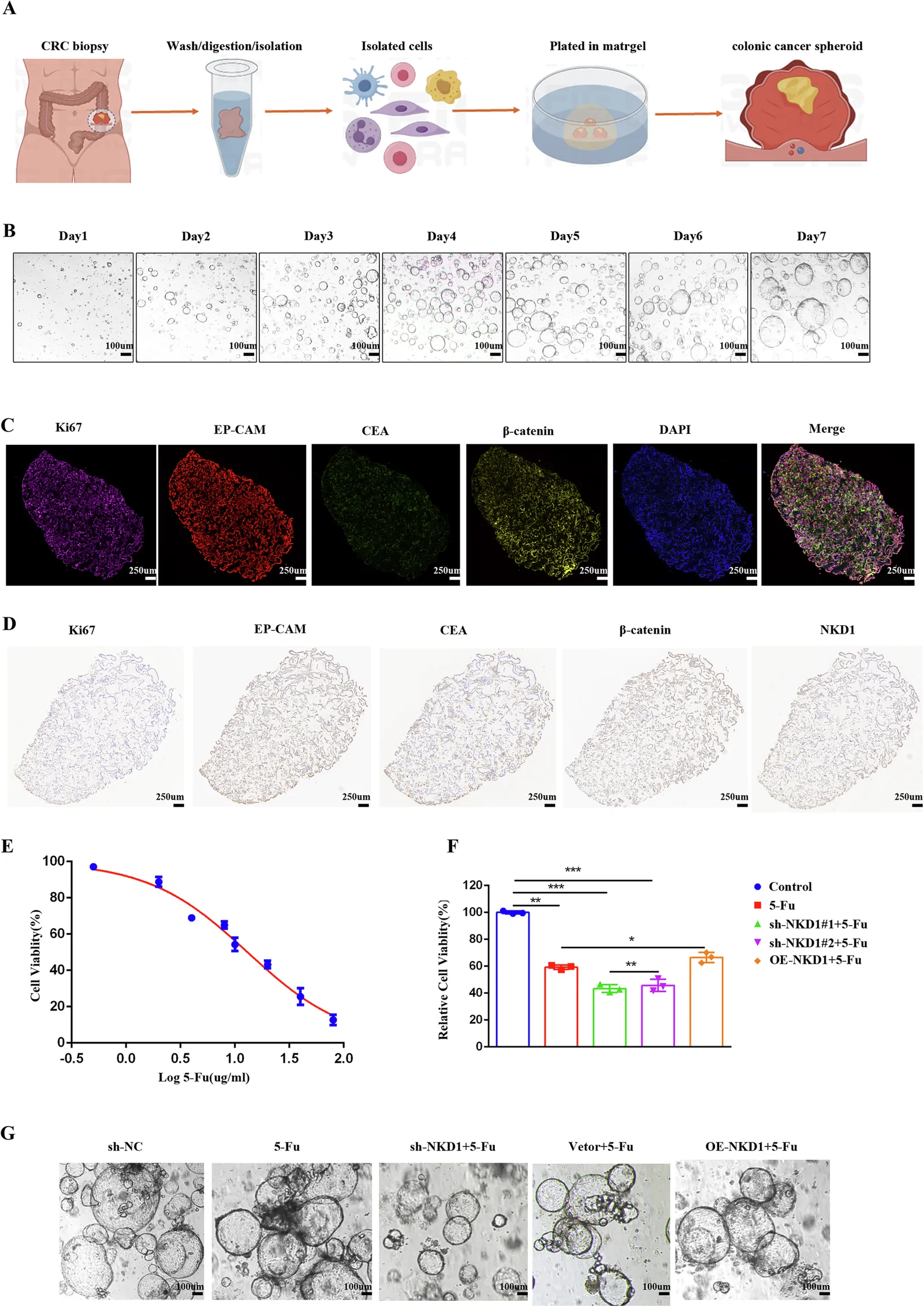
The role of NKD1 in 5-FU resistance was verified in CRC organoids. **A** Schematic diagram of organoid culture. **B** Growth images of organoid culture (bar = 100 μm). **C** Multiplex immunofluorescence staining of organoid-specific markers: Ki67 (pink), Ep-CAM (red), CEA (green), β-catenin (yellow), and DAPI (blue) (bar = 250 μm). **D** Immunohistochemical analysis of Ki67, CEA, Ep-CAM, β-catenin, and NKD1 expression in serial organoid sections (bar = 250 μm). **E** Effect of 5-FU on the viability of CRC organoid cells. **F** Effect of different treatment groups on CRC organoid cell viability. **G** Representative images showing the effects of different treatments on CRC organoid growth (bar = 100 μm; *p < 0.05, **p < 0.01, ***p < 0.001).

The workflow for CRC tissue dissociation and organoid establishment is illustrated in Fig. 6A. Organoid development was observed daily for seven days following resuscitation, and immunohistochemical analysis confirmed the expression of stemness markers, including CD44, Ep-CAM, and CEA. High NKD1 and Ki67 levels further indicated robust proliferative capacity (Fig. 6B–D). Following organoid maturation, a 5-FU sensitivity assay was performed. Various drug concentrations were applied, and the IC50 was determined to be 13.07 μg/mL after 7 days of treatment (Fig. 6E). Organoids were allocated into five groups: untreated control, 5-FU, two NKD1 knockdown + 5-FU, and OE-NKD1 + 5-FU. Cell viability was assessed utilizing the CellTiter-Lumi™ assay (Fig. 6F). As shown in Fig. 6G, sh-NKD1 lentivirus enhanced the response of organoids to 5-FU.

### NKD1 promoted CRC progression via activation of the β-Catenin pathway

**Fig. 7:**
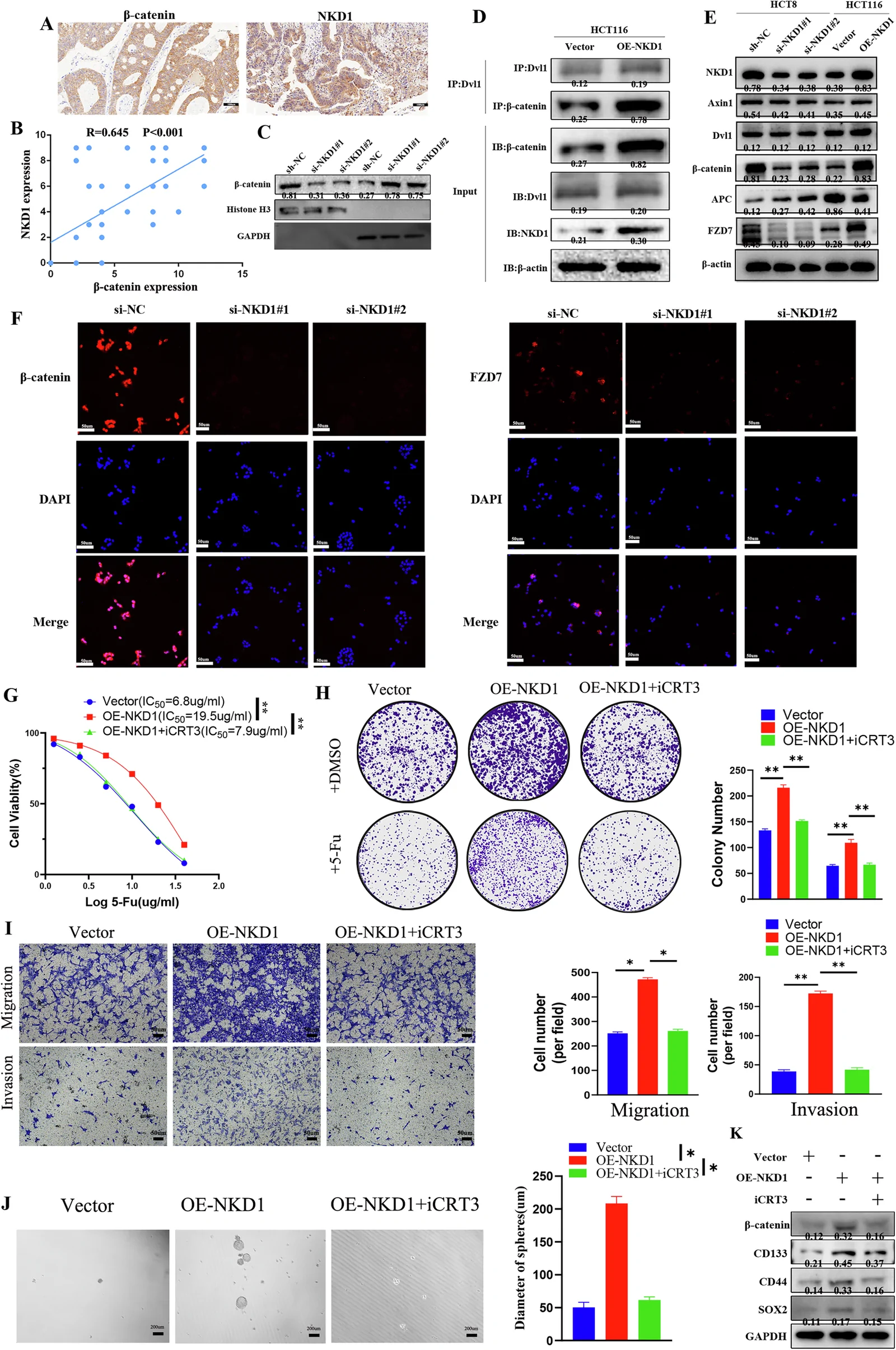
NKD1 promoted CRC progression by activating the β-catenin signaling. **A** Representative images of NKD1 and β-catenin expression in CRC tissue microarray (bar = 100 μm). **B** Pearson analysis showing a positive correlation between NKD1 and β-catenin expression based on IHC scores. **C** Nucleoplasmic isolation WB detecting β-catenin expression in NKD1 knockdown cells. **D** Co-IP results show that NKD1 promotes the direct binding of Dvl and β-catenin in vivo, activating β-catenin. **E** WB analysis of Wnt pathway-related proteins after NKD1 upregulation and downregulation. **F** Downregulation of NKD1 expression reduces β-catenin and FZD7 expression in HCT8 cells (bar = 50 μm). **G** HCT116 cells after overexpressing NKD1 levels (with or without iCRT3) were treated with different dosages of 5-Fu for 72 h, and cell viability was evaluated using CCK-8 assays. **H** Colony formation assays evaluated the effect of overexpressing NKD1 levels on the proliferative capacity of HCT116 cells (with or without iCRT3) in the presence or absence of 5-Fu. **I** Representative images of transwell migration and invasion assay in NKD1-overexpressing HCT116 cells with or without iCRT3 (50 μM) for 24 h. Scale bar: 50 μm. **J** Representative images of tumor sphere formation in NKD1 overexpressing HCT116 cells with or without iCRT3 for 24 h. The data represent a comparison of tumor sphere diameters among the groups (Right). Scale bar: 200 μm. **K** Western blot analysis of cancer stem cell markers and β-catenin protein expression levels in NKD1-overexpressing HCT116 cells with or without iCRT3 for 24 h. Results are expressed as mean ± standard deviation (SD). * indicates *P* < 0.05; ** indicates *P* < 0.01.

To investigate the mechanisms by which NKD1 promotes CRC initiation and progression and resistance to 5-FU, we analyzed NKD1 enrichment in TCGA CRC sequencing data utilizing Gene Set Enrichment Analysis (GSEA). The results indicated that elevated NKD1 expression was enriched in two Wnt signaling pathways. Linear regression analysis of the GEPIA dataset demonstrated a positive correlation between NKD1 and key Wnt pathway activators, including β-catenin, LRP6, LGR5, and Axin1, and negatively correlated with APC, FZD1, and FZD5 (Supplementary Fig. S4A, B). Additionally, TMA IHC analysis demonstrated that β-catenin expression levels correlated with tumor T and AJCC stages, and exhibited a strong positive association with NKD1 expression (Fig. 7A, B, Supplementary Fig.S4C–E). These findings suggest that NKD1 facilitates CRC progression by activating the Wnt/β-catenin axis. Although in Drosophila, NKD1 targets the globular domain of Disheveled (Dvl) to inhibit Wnt signaling, we hypothesized a regulatory role for NKD1 in stabilizing the β-catenin–Dvl complex in CRC. Nuclear-cytoplasmic fractionation further demonstrates that NKD1 knockdown suppresses the nuclear accumulation of β-catenin, suggesting impaired transcriptional activation (Fig. 7C). To investigate whether NKD1 affects the binding affinity between Dvl and β-catenin, we performed co-immunoprecipitation (Co-IP) assays. Using equal amounts of anti-Dvl antibody and recombinant protein A-agarose beads in the IP samples, we observed that NKD1 overexpression enhanced the interaction between Dvl and β-catenin, indicating that NKD1 is essential for the binding of Dvl to β-catenin. Furthermore, the Co-IP results demonstrated that NKD1 overexpression significantly upregulated β-catenin expression in cells (Fig. 7D). Western blot (WB) analysis further confirmed that NKD1 knockdown or overexpression regulated the expression levels of β-catenin, FZD7, and APC, while exerting no significant effect on AXIN1 and DVL1 (Fig. 7E). Given that APC protein abundance was altered following NKD1 modulation, we further investigated whether NKD1 regulates APC protein stability. To further investigate the mechanism by which NKD1 regulates APC protein abundance, we next examined whether NKD1 affects APC stability and degradation. Endogenous co-immunoprecipitation assays demonstrated a physical interaction between NKD1 and APC in both HCT8 and HCT116 cells (Supplementary Fig. S6A), suggesting that APC may serve as a direct downstream target of NKD1. Cycloheximide (CHX) chase assays revealed that NKD1 knockdown markedly prolonged APC protein stability in HCT8 and HCT15 cells, whereas NKD1 overexpression accelerated APC degradation in HCT116 cells (Supplementary Fig. S6B). Furthermore, treatment with the proteasome inhibitor MG132 restored APC protein levels in NKD1-overexpressing cells and diminished APC accumulation differences induced by NKD1 silencing (Supplementary Fig. S6C), indicating that APC degradation is proteasome-dependent. Consistently, ubiquitination assays demonstrated that NKD1 depletion reduced APC ubiquitination, while NKD1 overexpression enhanced APC ubiquitination levels (Supplementary Fig. S6D). Collectively, these findings indicate that NKD1 promotes APC degradation through a ubiquitin–proteasome-dependent mechanism. Consistently, immunofluorescence (IF) staining verified that modulation of NKD1 in cells affects β-catenin and FZD7 (Fig. 7F, Supplementary Fig. S4F, G). To further determine the role of the Wnt/β-catenin pathway in the function of NKD1 in CRC, we treated NKD1-overexpressing cells (HCT116) with the Wnt/β-catenin inhibitor iCRT3. We further confirmed through cell viability assays and colony formation experiments that iCRT3 significantly increased the sensitivity of NKD1-overexpressing HCT116 cells to 5-Fu (Fig. 7G, H). Transwell assays revealed that iCRT3 significantly reduced the migration and invasion abilities of CRC cells in NKD1-overexpressing HCT116 cells (Fig. 7I). Next, tumor sphere formation assays demonstrated that iCRT3 significantly diminished the sphere formation ability of NKD1-overexpressing HCT116 cells (Fig. 7J). Similarly, the elevated protein levels of β-catenin, CD44, CD133 and SOX2 due to NKD1 overexpression were reduced by iCRT3 (Fig. 7K). These data suggest that the Wnt/β-catenin signaling pathway plays a critical role in the enhancement of CRC stemness expression induced by NKD1, which contributes to 5-Fu resistance.

### Lentivirus-mediated NKD1 silencing inhibits colorectal tumor growth in patient-derived xenografts model

**Fig. 8:**
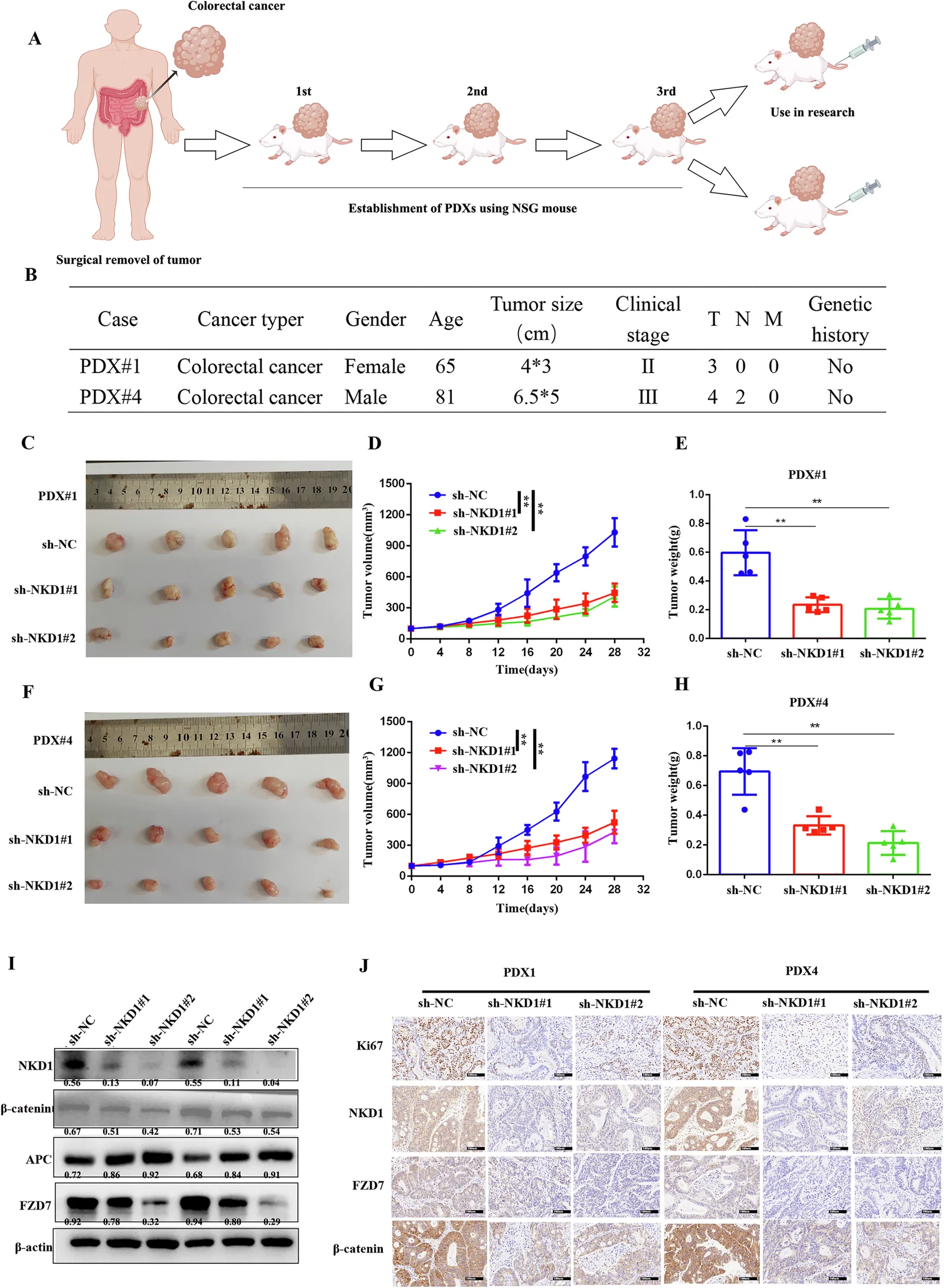
Lentivirus-mediated NKD1 silencing inhibits colorectal tumor growth in patient-derived xenografts model. **A** Workflow for establishing the PDX model. **B** Clinicopathological features of colorectal cancer patients in PDX models. **C**, **F** Representative images of PDX xenografts from subcutaneous tissue of NSG mice. **D**, **G** Growth volume of two PDX xenografts. **E**, **H** Final tumor weight of two PDX xenografts. **I** Expression of β-catenin, APC, and FZD7 after NKD1 knockdown. **J** IHC detection of Ki67, NKD1, FZD7, and β-catenin expression in different PDX model groups (bar = 100 μm). Results are expressed as mean ± standard deviation (SD). * indicates *P* < 0.05; ** indicates *P* < 0.01.

To assess the involvement of NKD1 in CRC progression in vivo, PDX were intratumorally injected with lentivirus to knock down NKD1 expression (Fig. 8A). NKD1 expression levels were confirmed by Western blotting, and PDX#1 and PDX#4, which showed elevated NKD1 expression, were selected to establish the PDX models (Fig. S5). Clinical information for PDX#1 and PDX#4 is presented in Fig. 8B. Lentiviral vectors carrying NKD1-specific shRNA were intratumorally administered to silence NKD1 expression. Tumor growth analysis showed that NKD1 knockdown significantly reduced tumor volume, final tumor weight, and average body weight compared to control groups (shNC) (Fig. 8C–H). Representative images of tumor suppression are shown Fig. 8C, F. Given that NKD1 oncogenicity in CRC is closely linked to the Wnt signaling, we evaluated downstream pathway activity in tumor tissues. Western blot and IHC analysis revealed marked reductions in NKD1, β-catenin, FZD7, and Ki67 expression following NKD1 silencing, consistent with reduced tumor burden (Fig. 8I, J). These findings underscore the role of NKD1 as a driver of CRC growth and indicate that lentivirus-mediated knockdown effectively impairs tumor progression via modulation of the Wnt/β-catenin axis in vivo.

## Discussion

NKD1, first identified in *Drosophila melanogaster*, is encoded by 10 exons located on human chromosome 16q12 [14]. By binding to the disheveled homology (Dvl) domain of Dishevelled proteins, NKD1 promotes their degradation, preventing the aberrant activation of the canonical Wnt/β-catenin pathway and suppressing tumorigenesis [15]. Downregulation of NKD1 has been observed in breast, lung, and osteosarcoma tissues, where it serves as a critical Wnt pathway antagonist [16–18]. However, its expression pattern varies in other cancers. For example, Arend Koch and colleagues observed elevated NKD1 levels in hepatoblastoma but reduced expression in hepatocellular carcinoma [19, 20]. Additionally, NKD1 overexpression has been reported in pulmonary papillary adenocarcinoma [21]. Notably, increased NKD1 expression relative to healthy tissues has been identified in certain murine intestinal tumor models, indicating its potential as a biomarker for tumor progression [22]. Consistent with a pro-tumorigenic role, our findings demonstrate that NKD1 activates the Wnt/β-catenin axis in CRC, promoting tumor growth and conferring 5-FU resistance.

The Wnt/β-catenin pathway is a highly conserved molecular cascade essential for embryonic development, stem cell maintenance, tissue homeostasis, and regeneration in adult organisms [23–26]. Activation of Wnt signaling stabilizes β-catenin, promoting the transcription of downstream target genes [27]. In most CRC patients, β-catenin is aberrantly activated, making it a key therapeutic target [28]. Notably, nuclear accumulation of β-catenin correlates with enhanced resistance to both chemotherapy and radiotherapy in advanced rectal tumors [29]. Pharmacological inhibition of β-catenin has been explored as a strategy to overcome drug resistance in CRC. For example, IC-2, a β-catenin pathway inhibitor, enhances 5-FU-mediated cytotoxicity and reduces expression of CSC markers in CRC cells [30]. Additionally, curcumin has been shown to inhibit CRC stemness by suppressing β-catenin signaling [31]. These findings underscore the therapeutic relevance of identifying upstream regulators that modulate Wnt/β-catenin signaling. Our work identifies NKD1 as one such regulator, demonstrating its role in β-catenin stabilization and nuclear translocation. This suggests that pharmacological inhibition of NKD1 could sensitize NKD1-high tumors to chemotherapeutic agents like 5-FU.

APC, a central negative modulator of the Wnt/β-catenin signaling, is crucial for assembling the β-catenin destruction complex, facilitating β-catenin degradation [32, 33]. In ~75% of CRC cases, truncation of the APC SAMP repeat sequence disrupts the APC-AXIN1 interaction [34], impairing the interaction between APC and β-catenin necessary for assembling the destruction complex [35]. Disruption of this interaction results in aberrant activation of β-catenin in CRC. Recent studies have shown that Let-7b-5p suppresses colon cancer cell proliferation and invasiveness by downregulating NKD1. NKD1 interacts with APC, promoting its degradation by inhibiting the deubiquitinase USP15 and preventing USP15 from binding to APC, activating β-catenin [36]. Supporting a regulatory relationship between NKD1 and APC, we found that NKD1 and APC expression levels are inversely correlated in our CRC models. Consistently, experimental upregulation of NKD1 decreased APC protein, while NKD1 knockdown increased it. To further clarify the mechanistic relationship between NKD1 and APC, we performed additional biochemical analyses. Co-immunoprecipitation assays demonstrated a physical interaction between NKD1 and APC, suggesting that APC may represent a direct intracellular target of NKD1. Moreover, cycloheximide chase experiments revealed accelerated APC protein degradation following NKD1 overexpression, indicating reduced APC stability. Ubiquitination assays further showed that NKD1 promotes APC ubiquitination, supporting a model in which NKD1 facilitates APC proteasomal degradation. These findings extend beyond correlative observations and suggest that NKD1 contributes to canonical Wnt activation through destabilization of the β-catenin destruction complex.

The FZD protein family, characterized by seven transmembrane domains, is crucial in mediating Wnt signaling [37]. In mammals, the family consists of ten members, FZD1 through FZD10 [38]. Each FZD protein can bind to one or more of the 19 Wnt ligands, activating the canonical and non-canonical Wnt signaling axis [39, 40]. Among them, FZD7 is frequently upregulated and has been associated with metastatic dissemination, advanced tumor stages, and unfavorable prognosis [41]. In gastric and esophageal malignancies, FZD7 facilitates epithelial-mesenchymal transition, thereby enhancing invasion and metastasis [42]. Additionally, the downregulation of FZD7 impairs the invasive and metastatic potential of CRC cells. We investigated whether NKD1 influences this key receptor. Our data show that NKD1 knockdown suppresses FZD7 protein expression. This, together with its effect on APC, indicates that NKD1 activates Wnt/β-catenin signaling in CRC through a multi-faceted mechanism involving both receptor and intracellular complex regulation. Notably, NKD1 has been reported to possess PDZ-binding activity, suggesting that it may participate in receptor-associated protein interactions involved in FZD7-mediated Wnt signaling. In addition to intracellular regulation of APC stability, NKD1 may also influence Wnt signaling at the receptor level. We observed that NKD1 positively regulates FZD7 expression in CRC cells, suggesting a potential role in enhancing upstream Wnt responsiveness. Although direct interaction between NKD1 and FZD7 has not yet been experimentally validated, previous studies have demonstrated that PDZ domain-associated adaptor proteins, such as GIPC2, can directly interact with FZD7 and promote Wnt/β-catenin signaling activation. Based on these findings, it is conceivable that NKD1 may participate in receptor-associated signaling complexes to facilitate pathway activation. Future studies involving structural interaction analysis or domain mapping will be required to verify this possibility.

In summary, NKD1 is upregulated in CRC, enhances 5-FU resistance, and is an independent predictor of clinical outcome. Mechanistically, NKD1 activates canonical Wnt signaling predominantly through APC destabilization and enhanced β-catenin stabilization, while receptor-associated regulation involving FZD7 may provide an additional layer of pathway amplification, thereby promoting CRC progression and 5-FU resistance. Critically, in vivo studies using a PDX model validated that targeting NKD1 overcomes 5-FU resistance. Collectively, these findings highlight NKD1 as a key modulator of chemoresistance and support its potential utility as both a predictive biomarker and a therapeutic target in combination strategies for CRC management.

## Supplementary information

Supplementary information figure legend

Supplementary Table

Supplemental Figure 1 Elevated mRNA and protein levels of NKD1 in colorectal cancer

Supplemental Figure 2 Knockdown and overexpression of NKD1 in colorectal cancer cell lines

Supplemental Figure 3 Colony formation analysis after si-NKD1 transfection in HCT8 cells with or without 5-FU treatment.

Supplemental Figure 4 GSEA, GEPIA, and clinical samples reveal NKD1’s correlation with the Wnt/β-catenin pathway

Supplemental Figure 5 NKD1 promotes the ubiquitinated degradation of APC

Supplemental Figure 6 NKD1 expression in 6 PDX models.

Original Data

## Data Availability

Data is available upon reasonable request. All data are available from the corresponding author upon request.
