# Supplementary material for: NKD1 promotes the progression and 5-FU resistance of colorectal cancer via the Wnt/β-catenin signaling
Source: Cell Death Dis. 2026 Jul 10;17(1):861. doi: 10.1038/s41419-026-09077-2 (PMC13639040; doi:10.1038/s41419-026-09077-2)

Fig1I

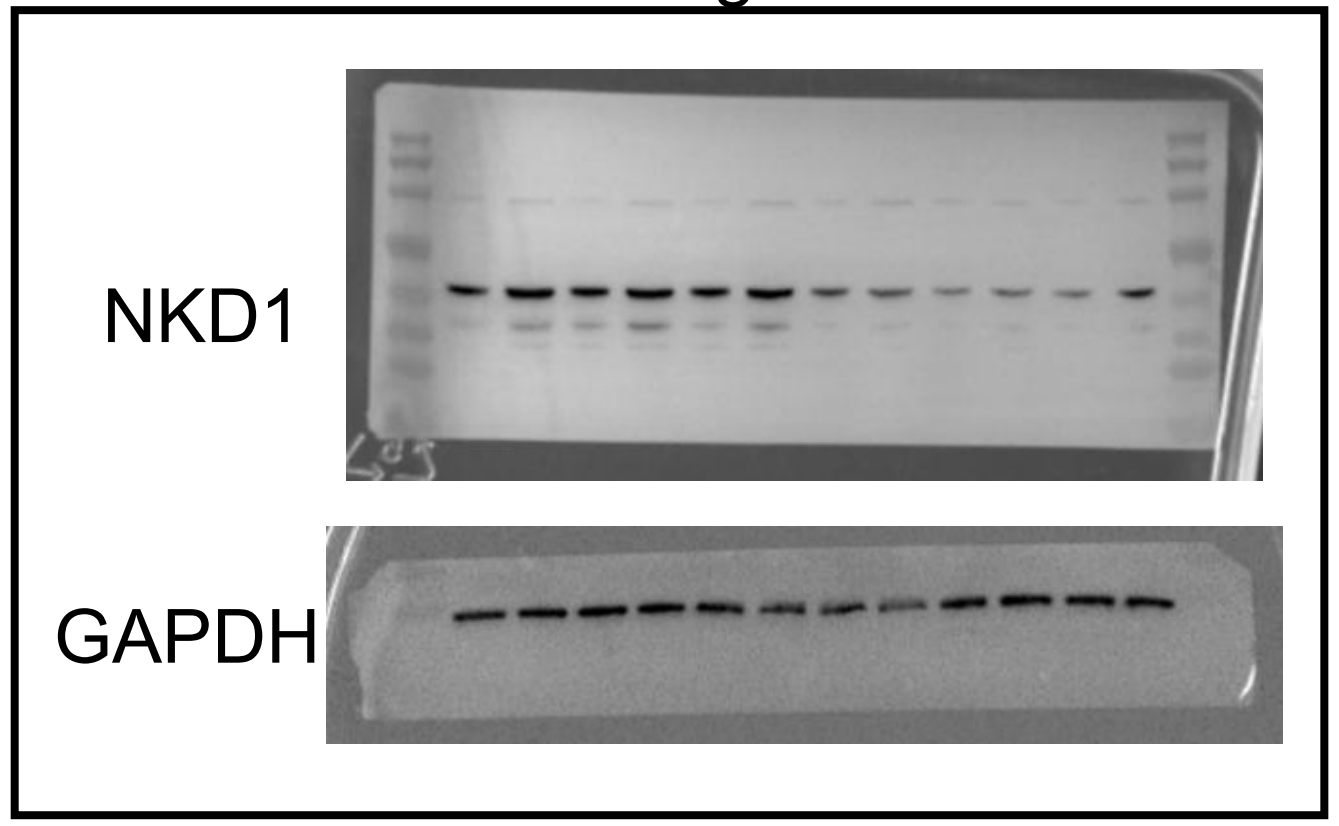

Fig2I

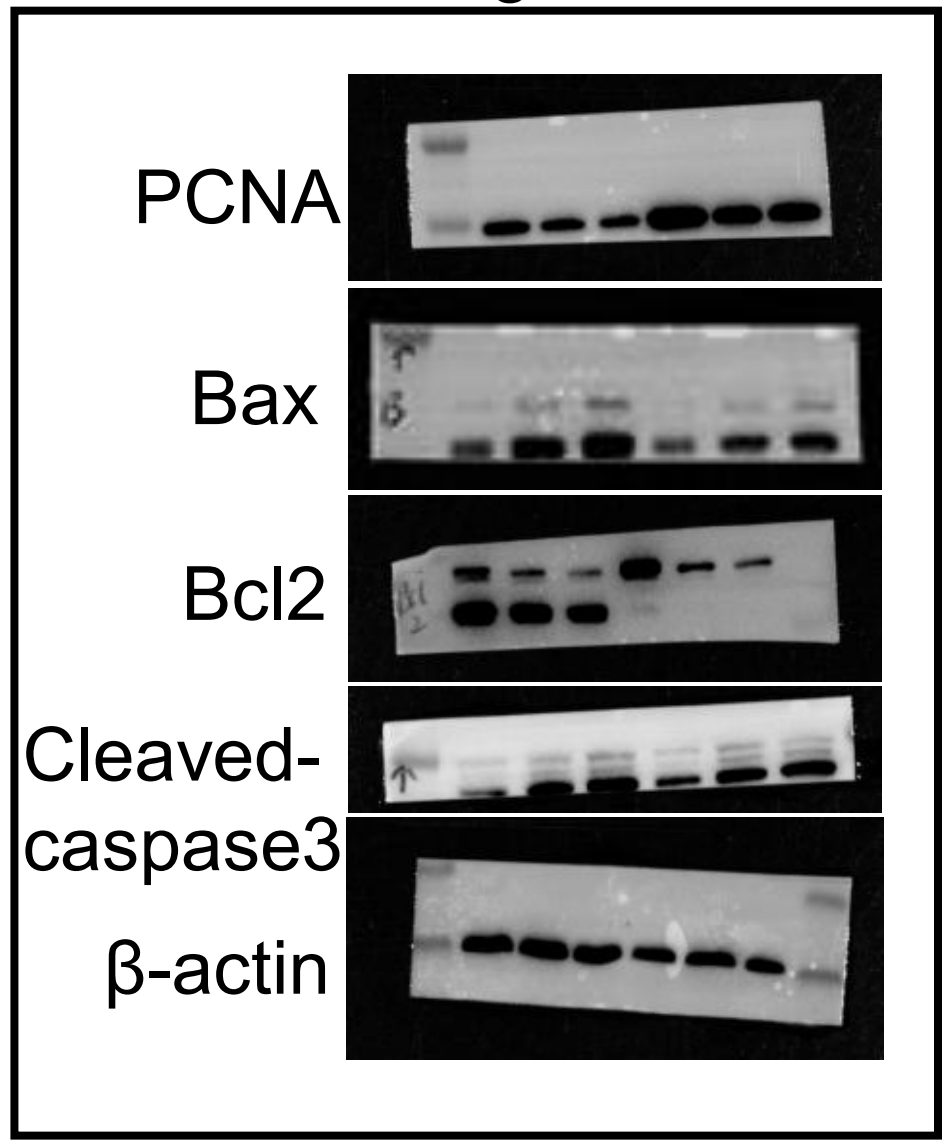

Fig2J

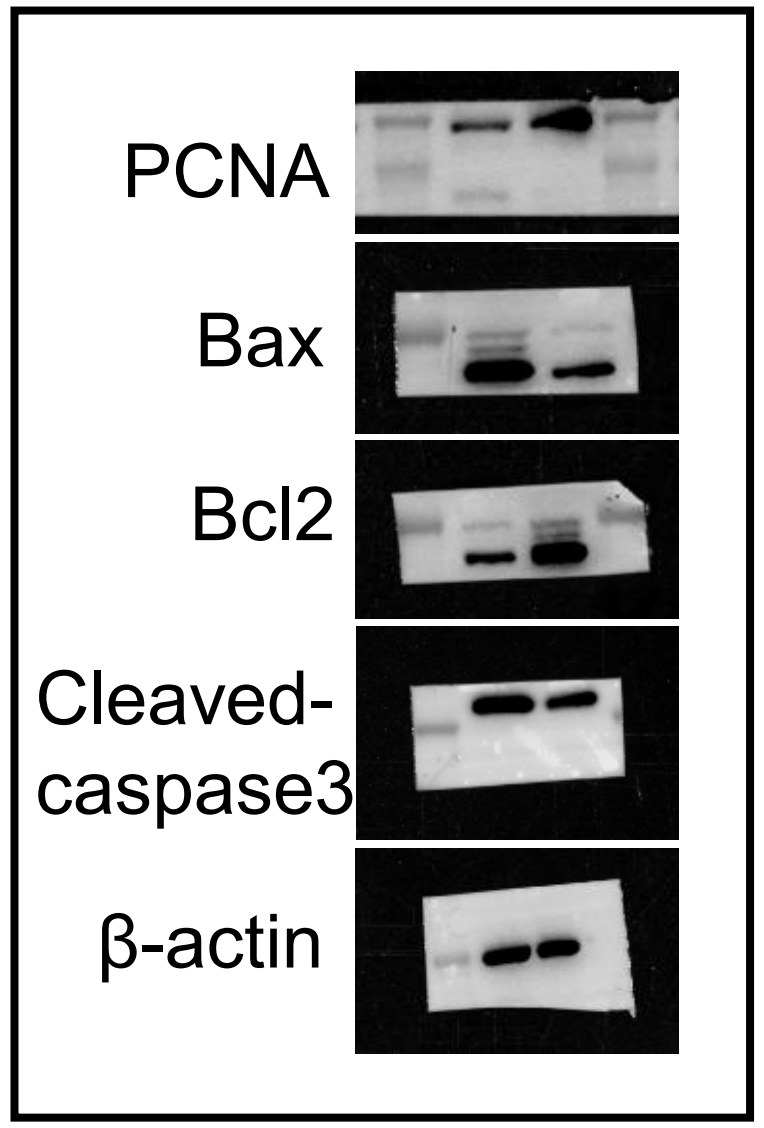

Fig3E

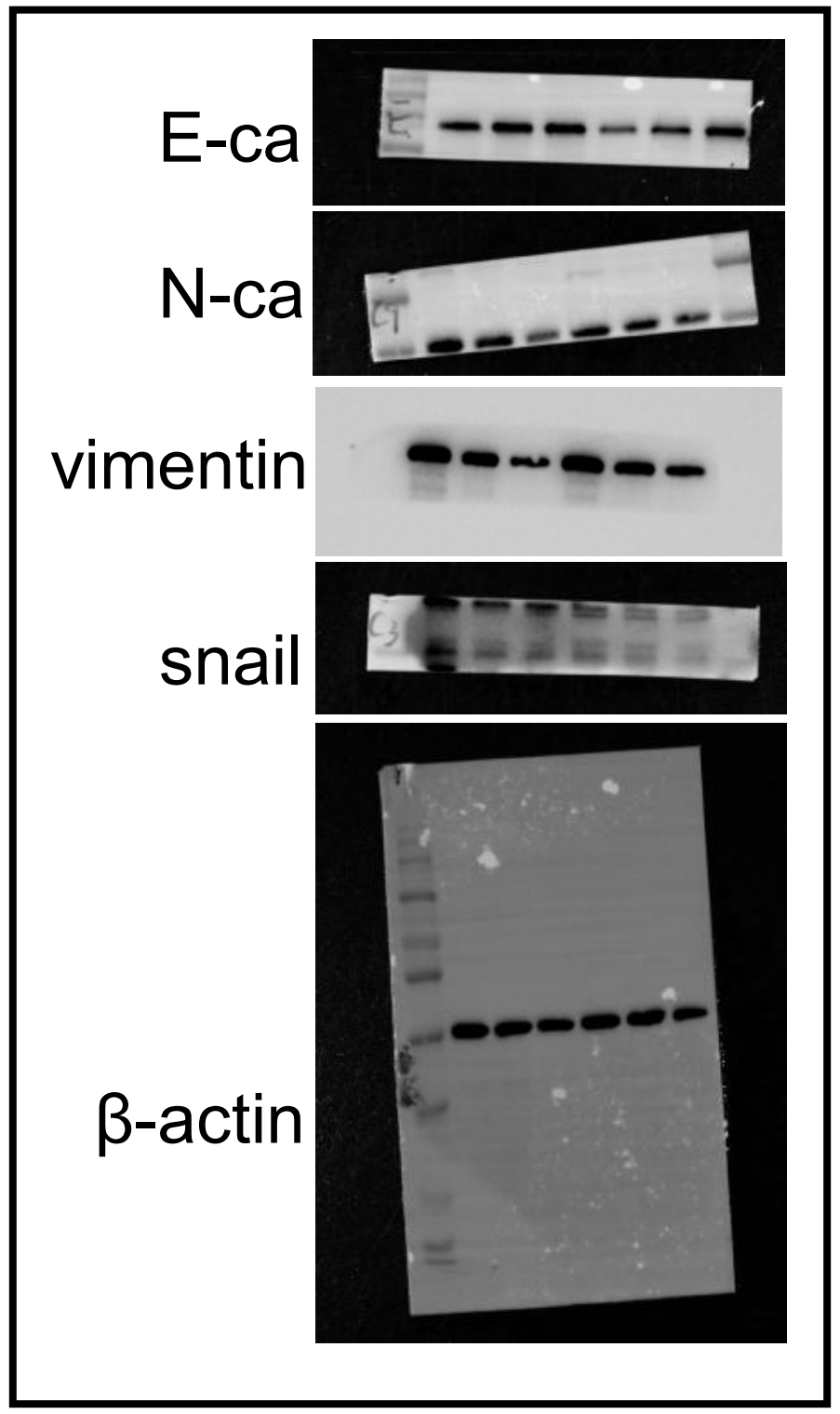

Fig3F

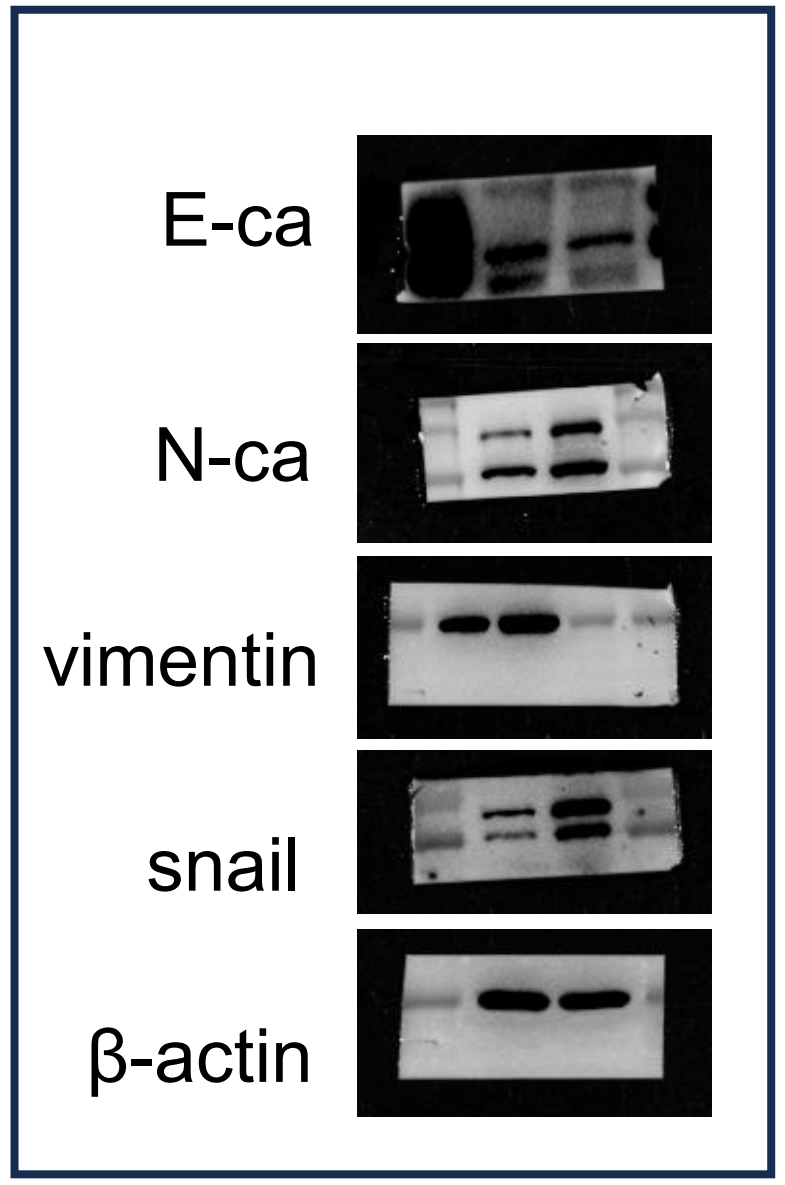

Fig4D

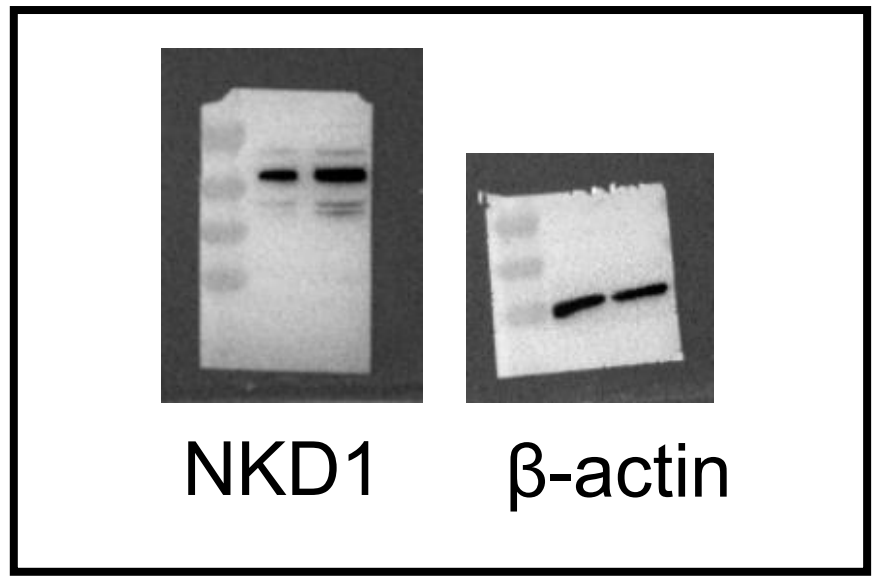

Fig5C

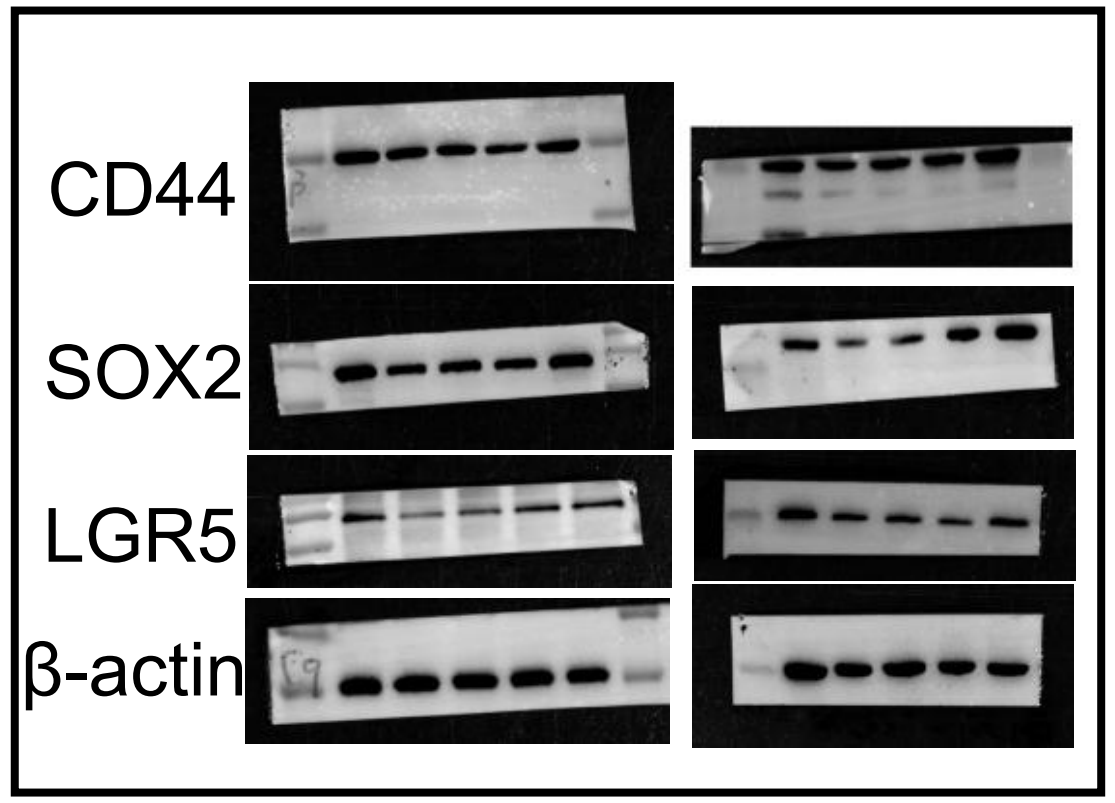

Fig7C

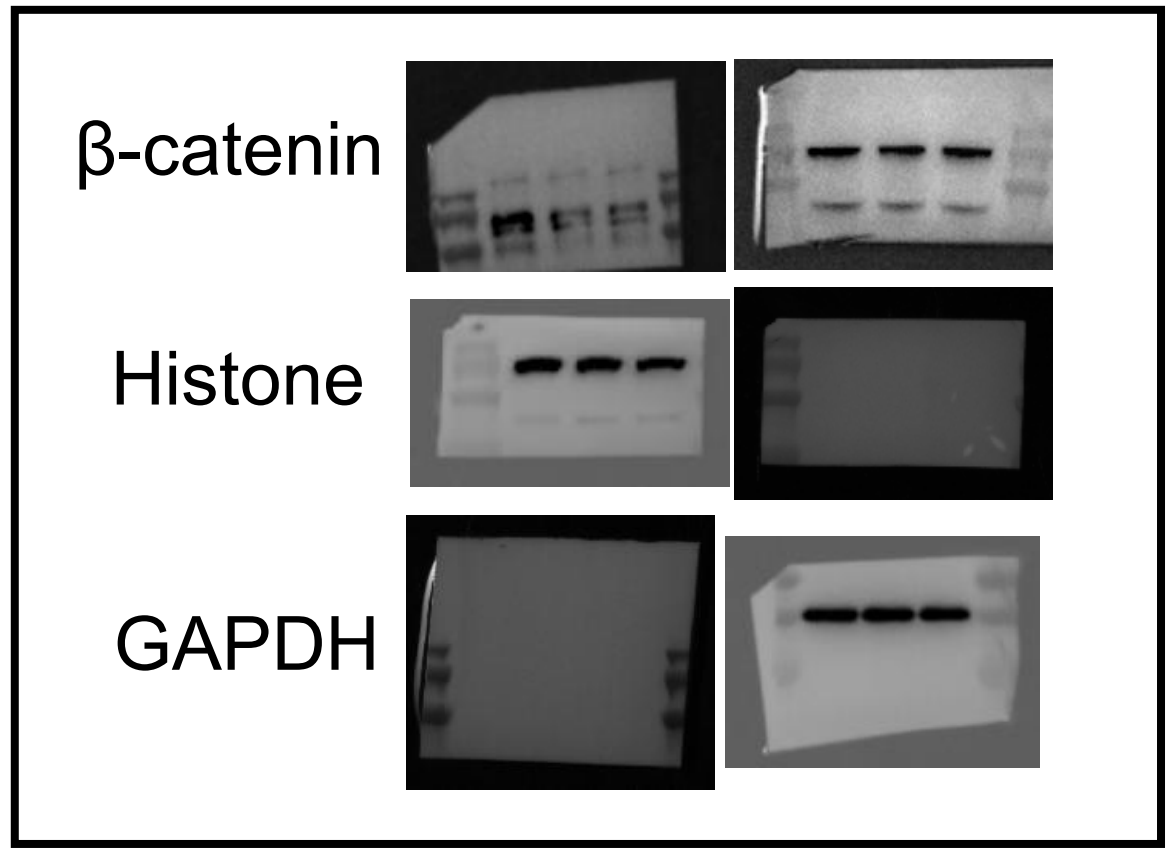

Fig7D

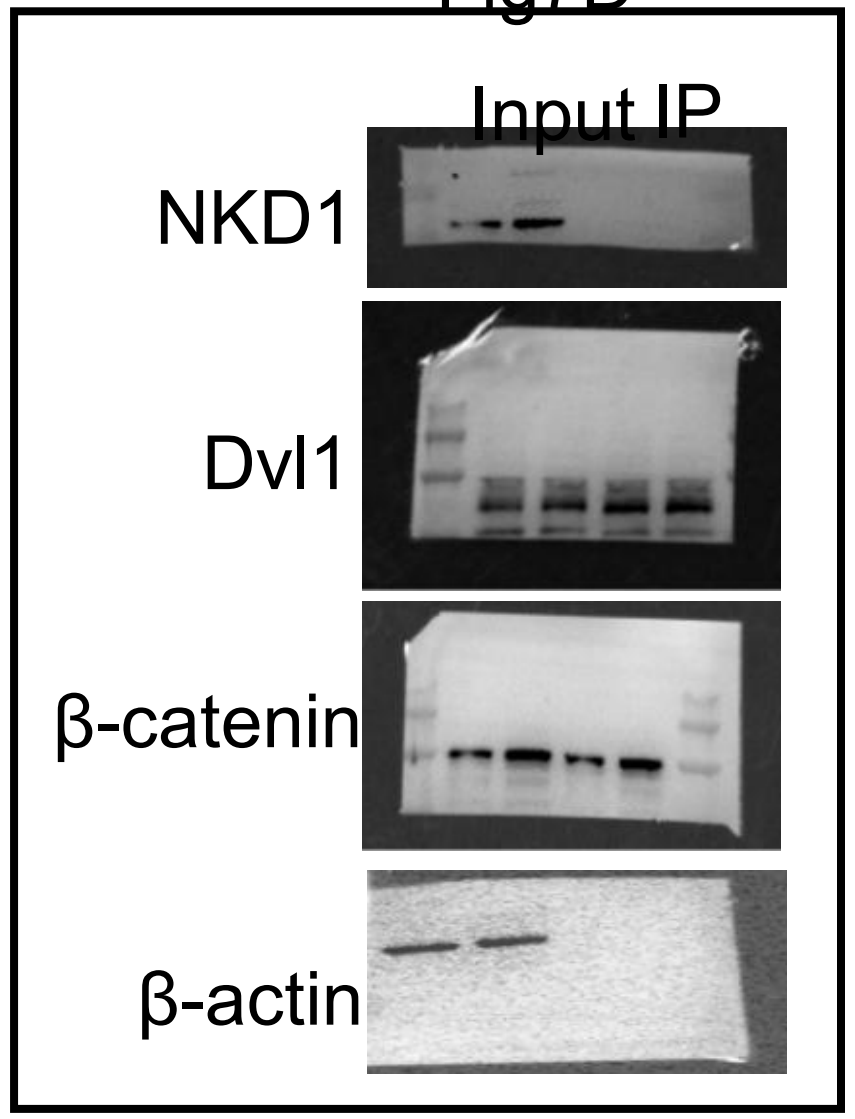

Fig7E

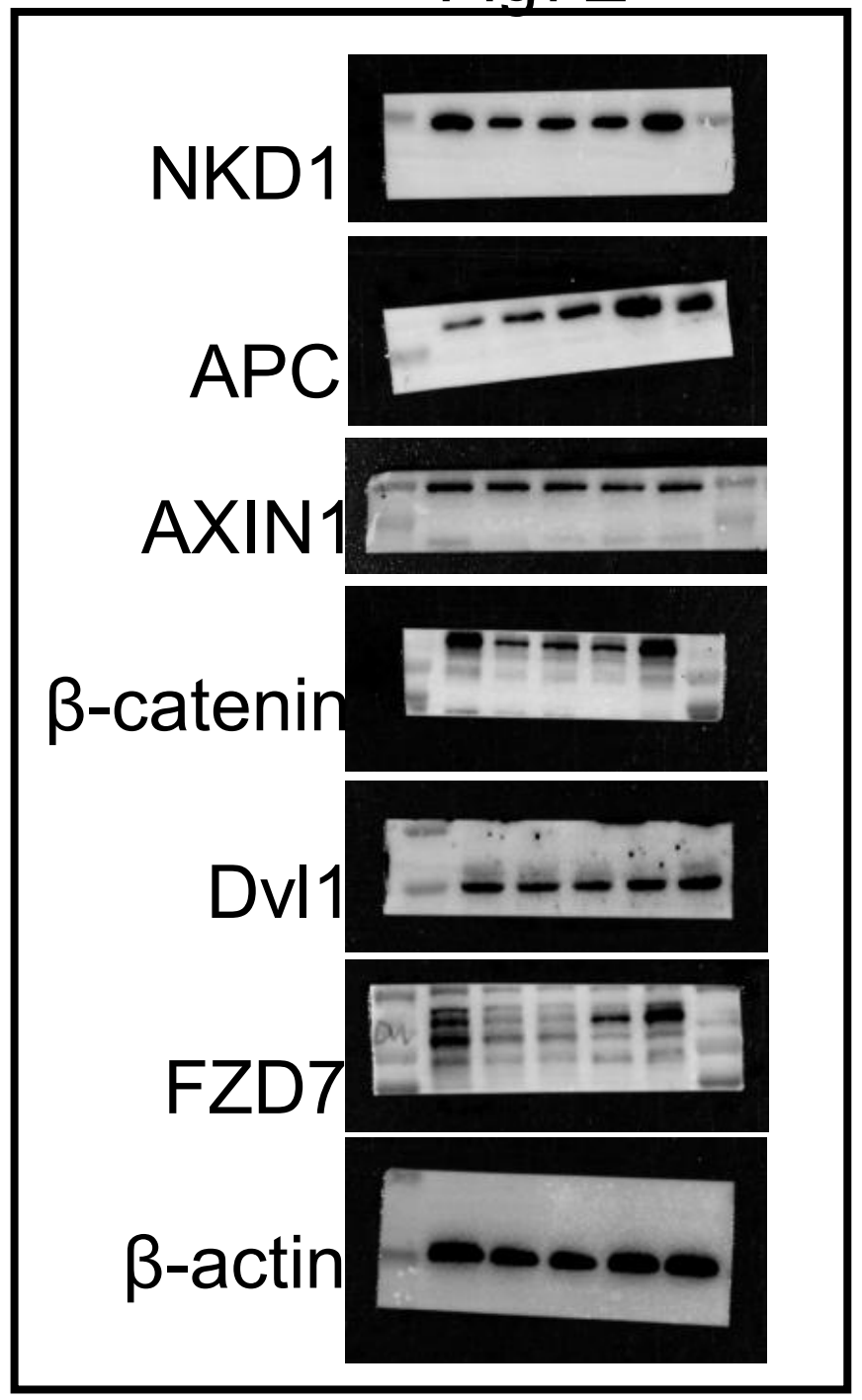

Fig7K

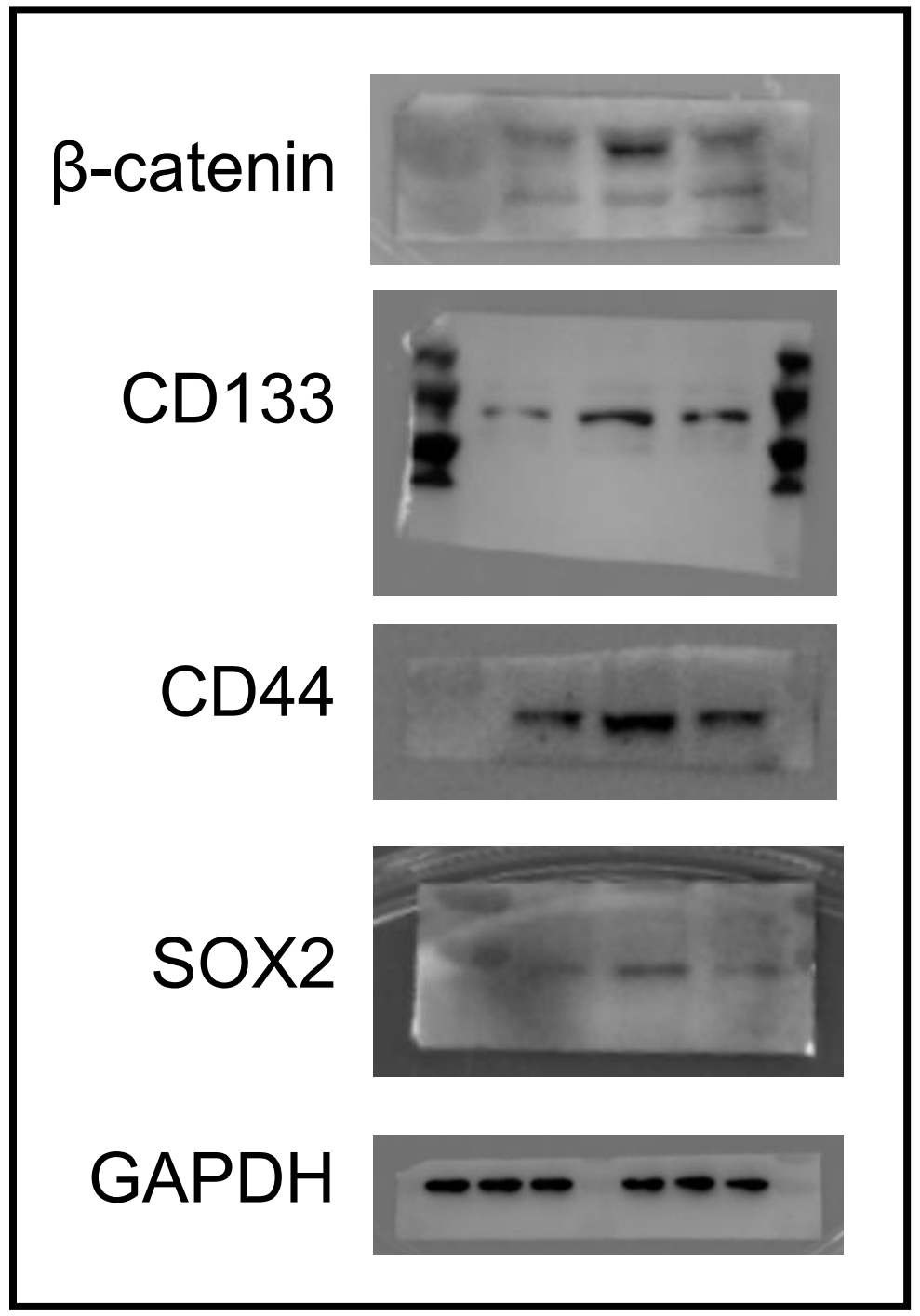

Fig8I

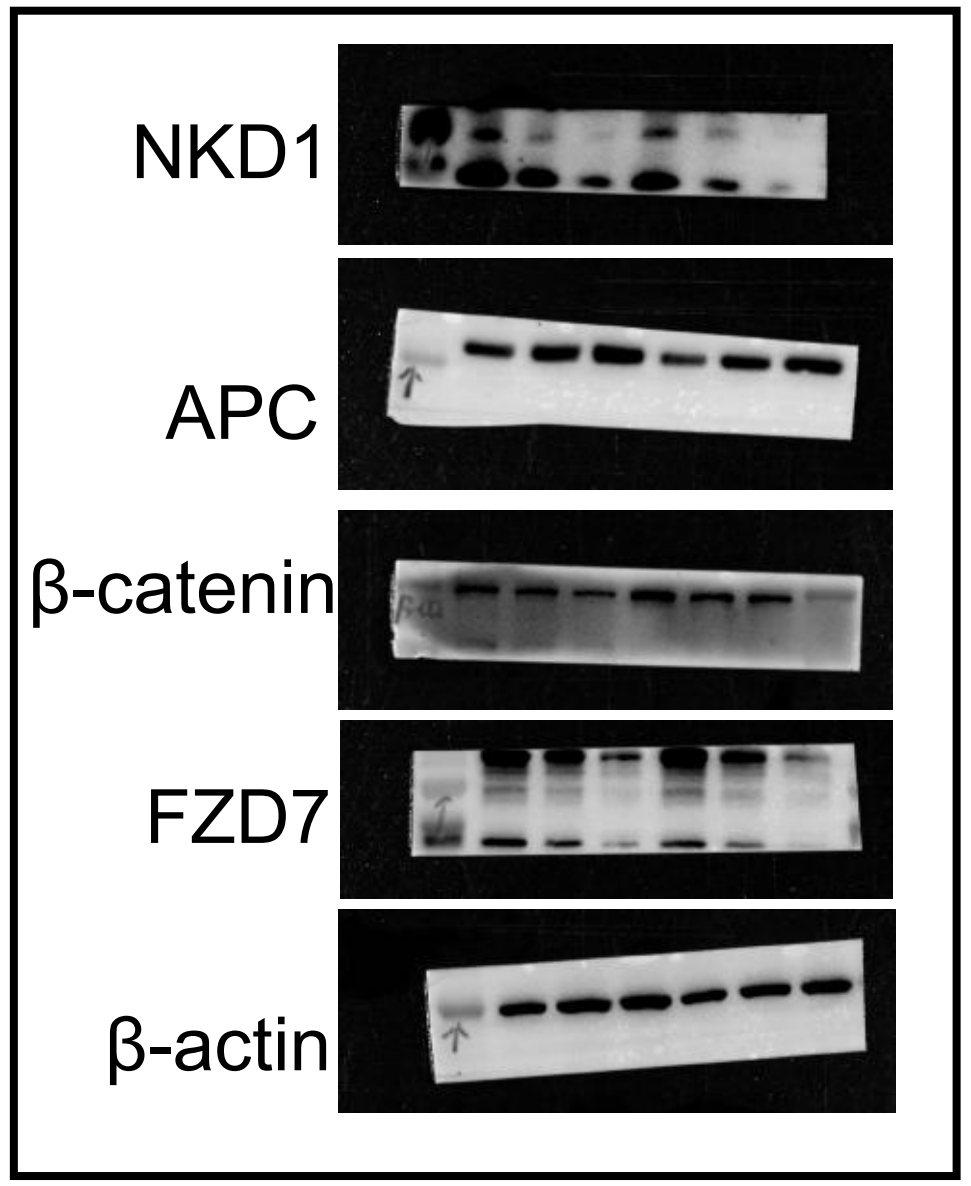

Supplement: Supplementary file 1 [file 41419_2026_9077_MOESM9_ESM.pdf]
