# Supplementary material for: NKD1 promotes the progression and 5-FU resistance of colorectal cancer via the Wnt/β-catenin signaling
Source: Cell Death Dis. 2026 Jul 10;17(1):861. doi: 10.1038/s41419-026-09077-2 (PMC13639040; doi:10.1038/s41419-026-09077-2)
Supplement: Supplementary file 2 [file 41419_2026_9077_MOESM1_ESM.docx]

**Supplemental Figure legends**

**Supplemental Figure 1. Elevated mRNA and protein levels of NKD1 in colorectal cancer**

A. Boxplots show the expression of six differentially expressed genes in normal and tumor samples from the COAD and READ datasets in the GEPIA database.

B. Expression of NKD1 mRNA in 11 colorectal cancer cells.

C. Expression of NKD1 protein in 10 colorectal cancer cells.

Results are expressed as mean ± standard deviation (SD). * indicates P < 0.05

**Supplemental Figure 2. Knockdown and overexpression of NKD1 in colorectal cancer cell lines**

A, B. The mRNA and protein expression of NKD1 in HCT8 cells after transfection with NKD1-siRNA.

C, D. The mRNA and protein expression of NKD1 in HCT15 cells after transfection with NKD1-siRNA.

E, F. The mRNA and protein expression of NKD1 in HCT116 cells after transfection with NKD1 overexpression plasmid.

G. Analysis of colony formation capacity in HCT8 and HCT15 cells after different NKD1 knockdown treatments.

H. EdU assay analysis of cell proliferation activity in HCT8 and HCT15 cell lines after NKD1 knockdown.S

I. Analysis of colony formation capacity in HCT116 cells following NKD1 overexpression.

J. EdU assay analysis of cell proliferation activity in HCT116 cells following NKD1 overexpression

K. Apoptosis analysis of HCT8 and HCT15 cell lines via flow cytometry after NKD1 knockdown.

L. Apoptosis analysis of HCT116 cells via flow cytometry following NKD1 overexpression.

M. Transwell assay analysis of cell migration and invasion capacity in HCT8 and HCT15 cell lines after NKD1 knockdown.

N. Transwell assay analysis of cell migration and invasion capacity in HCT116 cells following NKD1 overexpression.

O. Sphere formation assay analysis of sphere diameter in HCT8 and HCT15 cell lines under different NKD1 modulation treatments.

Results are expressed as mean ± standard deviation (SD). ns indicates P > 0.05; * indicates P < 0.05; ** indicates P < 0.01; *** indicates P < 0.001.

**Supplemental Figure 3.** Colony formation analysis after si-NKD1 transfection in HCT8 cells with or without 5-FU treatment.

Results are expressed as mean ± standard deviation (SD). ** indicates P < 0.01; *** indicates P < 0.001.

**Supplemental Figure 4. GSEA, GEPIA, and clinical samples reveal NKD1’s correlation with the Wnt/β-catenin pathway**

A. GSEA showed the enrichment of NKD1-related genes and signaling pathways.

B. Correlation between NKD1 and genes such as β-catenin, LRP6, LGR5, Axin1, APC, FZD1 and FZD5.

C. Representative images of β-catenin expression in colorectal cancer and corresponding adjacent normal tissues.

D. Comparison of β-catenin expression in T2 and T4 CRC tissues (bar = 200 μm).

E. Comparison of β-catenin expression in stage II and IV CRC tissues (bar = 200 μm)

F, G. Analysis of the mean fluorescence intensity (MFI) of β-catenin and FZD7 in different treatment groups (n=3 per group).

Results are expressed as mean ± standard deviation (SD). * indicates P < 0.05; ** indicates P < 0.01; *** indicates P < 0.001.

**Supplemental Figure 5.** **NKD1 promotes the ubiquitinated degradation of APC**

A. Co-immunoprecipitation of endogenous NKD1 and APC in HCT8 and HCT16 cells.

B. The control and NKD1‑knockdown cells, as well as the NKD1‑overexpressing cells, were treated with CHX (100 μg/ml) for the indicated time gradients. Western blot was used to detect protein levels (left panel). APC protein levels were quantified by ImageJ (right panel).

C. The control and NKD1‑knockdown cells, as well as the NKD1‑overexpressing cells， were pre-treated with DMSO and MG132 (25 µM) for 10 h. Then protein levels of APC and GAPDH were measured via western blot.

D. The control and NKD1‑knockdown cells, as well as the NKD1‑overexpressing cells，were incubated with MG132 (25 µM) for 10 h. The ubiquitination levels of APC were then detected by western blot.

**Supplemental Figure 6.** NKD1 expression in 6 PDX models.
