# Supplementary material for: NKD1 promotes the progression and 5-FU resistance of colorectal cancer via the Wnt/β-catenin signaling
Source: Cell Death Dis. 2026 Jul 10;17(1):861. doi: 10.1038/s41419-026-09077-2 (PMC13639040; doi:10.1038/s41419-026-09077-2)
Supplement: Supplementary file 3 [file 41419_2026_9077_MOESM2_ESM.docx]

Table S1. Primer sequences

| Genes | Sequences (5’-3’) |
| --- | --- |
| NKD1 | F: GATGGAGAGAGTGAGCGAAC |
|  | R: TAGATGGTGTGCAGCAAGCT |
| GAPDH | F: GGGAAGGTGAAGGTCGGAGT |
|  | R: GGGGTCATTGATGGCAACA |
| CD44 | F: GCAGGTATGGGTTCATAGAAGG |
|  | R: GTGTTGGATGTGAGGATGT |
| SOX2 | F: ATGCACAACTCGGAGATCAG |
|  | R: TTTATAATCCGGGTGCTCCTTC |
| LGR5 | F: TCTGAATGGTGCCTCACAAATA |
|  | R: ACGGTTTGAGGAAGAGATGAG |

Table S2. siRNA Primer sequences

| Genes | Sequences (5’-3’) |
| --- | --- |
| siNKD1-3 | F: GCGUAGAΜGAGAACAUCGA |
|  | R: UCGAΜGUUCUCAUCUACGC |
| siNKD1-2 | F: GGAGCGTCCTTGTCAATCA |
|  | R: ΜGAUΜGACAAGGACGCUCC |
| siNKD1-1 | F: GCUΜGCΜGCACACCAUCUA |
|  | R: UAGAΜGGΜGΜGCAGCAAGC |

Table S3. shRNA Primer sequences

| Genes | Sequences |
| --- | --- |
| shNKD1-1  shNKD1-2  sh-NKD1-3  sh-NKD1-4 | CCGGCCATTGCGTAGATGAGAACATCTCGAGATGTTCTCATCTACGCAATGGTTTTTT |
|  | CCGGCACCCTGTATGACTTTGACAACTCGAGTTGTCAAAGTCATACAGGGTGTTTTTT |
|  | CCGGAGCAAGATGCTGCGGGTAAAGCTCGAGCTTTACCCGCAGCATCTTGCTTTTTTT |
|  | CCGGCCACTTAAACAAGCGTGGTTTCTCGAGAAACCACGCTTGTTTAAGTGGTTTTTT |

Table S4. Primer Design for NKD1 Overexpression Fragment

| Genes | Sequences (5’-3’) |
| --- | --- |
| PC-h-NKD1-E/B-F  PC-h-NKD1-E/B-R | GGAGACCCAAGCTGGCTAGTTGAATTCGCCACCATGGGGAAACTTCACT |
|  | AGTCACTTAAGCTTGGTACCGAGGATCCTGTCTGGTAGAAGTGGTGGT |
